# Semiquantitative [¹²³I]FP-CIT SPECT metrics combined with machine learning improve clinical differentiation of Parkinson’s disease and atypical parkinsonian syndrome

**DOI:** 10.1007/s00259-026-08022-x

**Published:** 2026-07-03

**Authors:** Paula Fernández-Rodríguez, Pablo Franco-Rosado, Patricia Diaz-Galvan, Laura Muñoz-Delgado, Antonio Cristobal Luque-Ambrosiani, Miguel Ángel Labrador-Espinosa, Jesús Silva-Rodríguez, Elena Ojeda-Lepe, Daniel Macías-García, Astrid Adarmes-Gómez, Silvia Jesús, Fátima Carrillo, Jose Antonio Lojo-Ramírez, David García Solis, Michel J. Grothe, Pablo Mir

**Affiliations:** 1 Servicio de Medicina Nuclear, Instituto de Biomedicina de Sevilla, Hospital Universitario Virgen del Rocío/CSIC/Universidad de Sevilla, Seville, 41013 Spain; 2 Unidad de Trastornos del Movimiento, Servicio de Neurología, Instituto de Biomedicina de Sevilla, Hospital Universitario Virgen del Rocío/CSIC/Universidad de Sevilla, Seville, 41013 Spain; 3 Centro de Investigación Biomédica en Red sobre Enfermedades Neurodegenerativas (CIBERNED), Instituto de Salud Carlos III, Madrid, 28029 Spain; 4 Departamento de Psicología Experimental, Universidad de Sevilla, Seville, 41018 Spain; 5 Department of Psychiatry and Neurochemistry, Institute of Neuroscience and Physiology, The Sahlgrenska Academy at the University of Gothenburg, Mölndal, 431 39 Sweden; 6 Reina Sofía Alzheimer Center, CIEN Foundation, ISCIII, Madrid, 28031 Spain; 7 Departamento de Medicina, Facultad de Medicina, Universidad de Sevilla, Seville, 41009 Spain

**Keywords:** [¹²³I]FP-CIT SPECT, Parkinson’s disease, Atypical parkinsonian syndromes, Dopamine transporter, Machine learning, In vivo biomarker

## Abstract

**Purpose:**

To evaluate whether semiquantitative striatal [¹²³I]FP-CIT SPECT-derived metrics improve clinical differentiation of degenerative parkinsonism using an integrated machine learning approach.

**Methods:**

This cross-sectional study included 487 patients with Parkinson’s disease (PD) and 219 with atypical parkinsonisms (APS), classified as progressive supranuclear palsy (PSP, *n* = 127), multiple system atrophy parkinsonian type (MSA-P, *n* = 37), multiple system atrophy cerebellar type (MSA-C, *n* = 12), and corticobasal degeneration (CBD, *n* = 43). All participants underwent a [¹²³I]FP-CIT SPECT. Striatal [¹²³I]FP-CIT uptake was quantified using anatomical (caudate, putamen, ventral striatum) and functional (limbic, executive, sensorimotor) parcellations to calculate specific binding ratios, asymmetry indices, and inter-regional ratios. Discriminative performance of each metric was evaluated using receiver operating characteristic (ROC) curves analyses. A random forest classifier integrating all semiquantitative metrics was trained and validated, enabling data-driven identification diagnostic pathways.

**Results:**

Caudate-to-putamen and sensorimotor-to-limbic inter-regional ratios showed the strongest discriminative performance (AUC up to 0.95) for differentiating PD from APS. The random forest achieved a 64% overall accuracy with high per-class specificities (> 84%) and revealed two diagnostic pathways. A lower caudate-to-posterior putamen ratio, primarily grouped PSP, CBD and MSA-C, where lower contralateral sensorimotor uptake pointing to PSP while higher values and ipsilateral caudate uptake subregion further distinguishing CBD from MSA-C. A higher caudate-to-posterior putamen ratio, included PD and MSA-P, where lower ipsilateral caudate uptake together with a higher sensorimotor-to-cognitive ratio mainly differentiate both groups.

**Conclusions:**

Integrating anatomical and functional [¹²³I]FP-CIT SPECT metrics within a machine learning framework enhances the clinical differentiation of degenerative parkinsonisms and supports [¹²³I]FP-CIT SPECT as a robust in vivo disease biomarker.

**Supplementary Information:**

The online version contains supplementary material available at https://doi.org/10.1007/s00259-026-08022-x.

## Introduction

Parkinsonian syndromes represent a complex spectrum of disorders characterized by bradykinesia and slowness of movement, which are mainly distinguished by their distinct underlying pathologies [1]. Although Parkinson’s disease (PD) is the most prevalent pathology, atypical degenerative parkinsonian syndromes (APS), including progressive supranuclear palsy (PSP), multiple system atrophy (MSA) and corticobasal degeneration (CBD), follow a more severe course and resist standard dopaminergic treatment. Accurate clinical differentiation between PD and APS is therefore highly relevant for effective clinical management and therapeutic decision-making [2].

However, differentiating PD from APS remains challenging as diagnosis still largely depends on clinical evaluation and is often inaccurate, even at specialist centers [3–5]. Autopsy studies reveal that approximately 80% of cases are diagnosed as PD, so APS cases are frequently misclassified [6]. Thus, reliable in vivo biomarkers are urgently needed to enable accurate diagnosis, guide treatment, and advance disease-specific therapeutics [7].

Cerebral molecular imaging with [¹²³I]FP-CIT SPECT is a key tool for assessing in vivo presynaptic dopaminergic function [8]. By quantifying dopamine transporter (DaT) density in the striatum, reflecting nigrostriatal integrity, this technique provides a robust biomarker of neurodegenerative parkinsonism [9]. In clinical practice, [¹²³I]FP-CIT SPECT is widely used to distinguish degenerative from non-degenerative causes of parkinsonism [10–12]. Characteristic patterns of striatal degeneration have been also described to distinguish between PD and other forms of APS: posterior putaminal asymmetric loss in PD, diffuse and symmetric involvement in PSP, rapid and widespread loss in MSA, and marked asymmetry in CBD. Furthermore, semi-quantitative approaches based on specific binding ratios, such as the putamen-to-caudate ratio have shown to provide additional information [13–17]. Some studies have implemented functional parcellations in [¹²³I]FP-CIT SPECT to enhance the characterization of dopaminergic loss patterns in PD and vascular parkinsonisms [18]. They used a connectivity-based atlas to divide the striatum into functional subregions according to their predominant projection to different cortical territories, such as limbic, executive and sensorimotor cortical regions [19, 20].

However, diagnostic application of [¹²³I]FP-CIT SPECT to discriminate different subtypes of APS from PD remains limited and findings are partly inconsistent [21–23]. These limitations underscore the need to move beyond visual reads or simple semi-quantitative metrics, that typically use a single striatal parcellation scheme, and to integrate neuroimaging data into advanced classification models that combine different striatal parcellations, binding ratios and related metrics. In this context, there is a growing interest in machine learning approaches applied to neuroimaging. In fact, machine-learning approaches have already shown promise for the differential diagnosis of parkinsonian syndromes, including the distinction of PD from vascular parkinsonism [12, 18] and the classification of degenerative parkinsonism subtypes based on semiquantitative ¹²³I-FP-CIT SPECT metrics derived from a conventional anatomical striatal parcellation scheme [17]. In this study, we sought to determine whether regionally detailed striatal metrics derived from [¹²³I]FP-CIT SPECT could improve the differential diagnosis of PD and atypical parkinsonian syndromes within a multiclass cohort including PSP, multiple system atrophy parkinsonian type (MSA-P), multiple system atrophy cerebellar type (MSA-C), and CBD. To this end, we combined anatomical and functional striatal parcellations to compare region-of-interest (ROI)–based [¹²³I]FP-CIT uptake values and derive specific binding ratios and asymmetry indices for each APS group relative to a reference cohort of PD patients. In addition, we explored disease-specific uptake patterns across APS subtypes to evaluate the discriminatory potential of [¹²³I]FP-CIT SPECT metrics for within-group differentiation. Moving beyond conventional univariate group comparisons, we developed and validated a machine learning–based classification model that integrates multidimensional [¹²³I]FP-CIT SPECT features to enable automated differentiation of neurodegenerative parkinsonian syndromes and to quantify the relative contribution of individual imaging biomarkers to overall diagnostic performance.

## Patients and methods

### Participants and clinical assessment

This retrospective cross-sectional study included patients with PD, PSP, MSA and CBD, recruited between 2006 and 2024 through the Movement Disorders Unit of the Hospital Universitario Virgen del Rocío (Seville, Spain). Considering that MSA presents two main subtypes, MSA-P and MSA-C, which differ in both clinical course and imaging characteristics [24], we considered them separately in our analyses. For the present study, we included patients from this cohort based on the availability of a [¹²³I]FP-CIT SPECT scan, which was acquired on average 4.5 ± 5.2 years after symptoms onset.

Our final study sample comprised 127 patients with PSP, 37 MSA-P, 12 MSA-C and 43 CBD, as well as 487 idiopathic PD patients. All patients’ medical records were carefully reviewed and patients were diagnosed by movement disorders specialists following the currently accepted diagnostic criteria for PD [25], PSP [26], CBD [27], and MSA [24].

Written informed consent was obtained from all alive participants during their clinical visits. For deceased patients, the ethics committees explicitly authorized the retrospective use of their imaging and clinical data without the need for additional consent. The study was conducted in accordance with the Declaration of Helsinki and approved by the Ethics Committee of the Hospital Universitario Virgen del Rocío (SICEIA-2025-00623) and by the Coordinating Committee for Biomedical Research Ethics of Andalusia (CCEIBA; S2300212).

## Neuroimaging acquisition and preprocessing

All images were acquired following a standardized imaging protocol for [¹²³I]FP-CIT SPECT (see Supplementary Material).

[¹²³I]FP-CIT SPECT images were processed using Statistical Parametric Mapping (SPM12; Wellcome Centre for Human Neuroimaging, Institute of Neurology, UCL, London, UK) implemented in MATLAB R2024a (MathWorks, Natick, MA, USA). Images were reoriented and normalized to the standard stereotactic Montreal Neurological Institute (MNI) space using an in-house [¹²³I]FP-CIT SPECT template [28]. The normalized images were resliced into a matrix of 91 × 109 × 91 voxels with a voxel size of 2 × 2 × 2 mm³. The specific binding ratio (SBR) for each voxel was calculated using the following formula: SBR = (voxel uptake − mean occipital lobe uptake)/mean occipital lobe uptake [29].

## Striatal [¹²³I]FP-CIT quantification

[¹²³I]FP-CIT levels were quantified as the mean SBR within each region of interest (ROI) in the left and right hemispheres. ROIs were defined according to two complementary striatal parcellation schemes. The first was a classical anatomical scheme differentiating the caudate, anterior putamen, posterior putamen, and ventral striatum bilaterally. Structurally, the putamen was further subdivided into anterior and posterior subregions using the anterior commissure as the reference point to enhance sensitivity to disease-specific patterns of nigrostriatal degeneration, particularly the posterior predominance observed in PD and related syndromes [30]. The second was a connectivity-based scheme derived from differential nigrostriatal pathway projections to cortical networks [20], identifying three functional subregions: a limbic-associated ROI (connected to the orbital gyrus, gyrus rectus, and subcallosal gyrus/ventral anterior cingulate), an executive-associated ROI (linked to the rostral superior and middle frontal gyri and dorsal prefrontal cortex), and a sensorimotor-associated ROI (associated with the rostral area 6, pre-supplementary motor area, frontal eye field, precentral gyrus, and parietal lobe). To better capture striatal regions involved in cognition, we also considered a “cognitive ROI” combining the limbic and executive territories [31, 32]. For both parcellation schemes, the more affected side was defined as the hemisphere with the lower posterior putamen SBR [33]. Final ROI values were then categorized as ipsilateral (I) or contralateral (C) relative to this more affected side. Bilateral ROI SBRs were also calculated as the average between contralateral and ipsilateral values. Additionally, asymmetry indices were calculated for each ROI using the following formula [10]: AI = [(contralateral ROI binding − ipsilateral ROI binding)/(contralateral ROI binding + ipsilateral ROI binding)] × 2 × 100. Finally, caudate-to-anterior putamen [Cau-Put (ant.)] and caudate-to-posterior putamen [Cau-Put (post.)] ratios, sensorimotor-to-limbic (Sen-Lim), sensorimotor-to-executive (Sen-Exe) and sensorimotor-to-cognitive (Sen-Cog) ratios were also calculated by dividing bilateral regional SBRs.

### Statistical analysis

Descriptive demographics are shown as mean (standard deviation) for normally distributed continuous variables or median (minimum-maximum) for non-normally distributed variables. Number and percentage were reported for categorical variables (sex). To assess group differences in striatal [¹²³I]FP-CIT SBR and derived metrics (i.e., binding ratios and asymmetry indices), a series of linear models were fitted for each [¹²³I]FP-CIT metric, including the clinical group as predictor and age, sex, and disease duration as covariates. Estimated marginal means (EMMeans) were computed for each group. Pairwise group comparisons were conducted on these EMMeans and corrected using the Benjamini–Hochberg method to control for multiple comparisons. For each contrast, 95% confidence intervals were calculated, and standardized effect sizes (Cohen’s *d*) were estimated. Receiver operating characteristic (ROC) curves were generated for [¹²³I]FP-CIT metrics in the anatomical and connectivity-based striatal ROIs. The area under the curve (AUC) was used to quantify the ability of each regional metric to discriminate PD from individual APS subtypes and, secondarily, to differentiate between APS subtypes. Optimal thresholds were determined using the Youden index, and 95% confidence intervals for AUC were computed using the DeLong method. Sensitivity, specificity, positive predictive value, and likelihood ratios (LR + and LR−) were calculated at the optimal threshold.

Finally, we used random forest analysis to develop and test a predictive model combining all [¹²³I]FP-CIT metrics to classify patients into PD or any of the APS (CBD, MSA-P, MSA-C, PSP). Random Forest was selected as it is well-suited to moderate-dimensional tabular ROI features, naturally captures non-linear relationships and feature interactions, provides interpretable variable importance measures, and has shown robust performance in small-sample neuroimaging classification settings compared to more complex alternatives [34]. The performance of the random forest classifier was evaluated using stratified 10-fold cross-validation repeated 10 times. Prior to modelling, the effect of disease duration on each [¹²³I]FP-CIT metric was removed using linear regression, with the regression model fitted exclusively on the training fold at each cross-validation iteration and the resulting coefficients applied to both the training and validation folds. This ensures that no information from the validation fold influences the preprocessing of the training data, while removing progression-related variance and preserving diagnosis-specific imaging patterns. Further details on the residualisation strategy are provided in the Supplementary Material. Furthermore, because the diagnostic groups were imbalanced (with a significant predominance of the PD group versus the other APS groups), we applied the Synthetic Minority Over-sampling Technique (SMOTE) [35] to generate synthetic examples of underrepresented groups within training folds, reducing bias toward the majority class. The RF was trained with 500 trees per fold (mtry = 3). Overall accuracy, macro-averaged balanced accuracy, and macro-averaged F1 score, together with class-specific sensitivity, specificity, and precision, were computed from aggregated out-of-fold predictions. Variable importance was averaged across all 100 folds. To provide an interpretable summary of the RF classifier, a single distilled decision tree was then fitted to the out-of-fold RF predictions using the same set of predictors.

A complementary analysis treating MSA as a single homogeneous group is also provided (see Supplementary Material. Tables S4-7. Figure S2). Finally, an additional complementary analysis is provided in which binding values are organized based on their native anatomical laterality (left vs. right hemisphere) rather than according to the laterality criterion used in the main analysis (see Supplementary Material. Table S8. Figure S3).

## Results

### Demographics and clinical features of patients

Demographics and clinical characteristics of the different patient groups are summarized in Table 1. Significant between-group differences were observed for age, and disease duration (both *p* < 0.001) whereas sex distribution did not differ significantly (*p* = 0.317). Patients with CBD (72.1 ± 6.5 years) and PSP (71.8 ± 7.1 years) were significantly older than those with MSA (MSA-P: 61.9 ± 8.9 years; MSA-C: 63.0 ± 5.7 years) and with PD (60.7 ± 10.6 years). Sex distribution also differed significantly, with all APS groups showing higher female representation compared to PD. The PD group had significantly longer disease duration (≃ 2.5 years longer) than all APS groups.

Descriptive statistics (mean ± SD) for [¹²³I]FP-CIT binding values and derived inter-regional ratios and asymmetry indices across both anatomical and functional striatal regions are provided in Table 1.

**Table 1:** Cohort characteristics

	CBD	MSA-*P*	MSA-C	PSP	PD	p*
n	43	37	12	127	487	
Sex, n (%) Female	18 (42%)	18 (49%)	5 (42%)	56 (44%)	177 (36%)	0.317
Age	72.1 (6.5)	61.9 (8.9)	63.0 (5.7)	71.8 (7.1)	60.7 (10.6)	< 0.001
Disease duration	2.7 (2.0)	2.2 (2.0)	2.5 (1.5)	2.4 (1.9)	5.6 (6.2)	< 0.001
SBR Ventral Striatum (I)	1.4 (0.5)	1.0 (0.4)	1.7 (0.5)	1.2 (0.5)	1.4 (0.5)	< 0.001
SBR Ventral Striatum (C)	1.5 (0.4)	1.1 (0.4)	1.6 (0.5)	1.3 (0.5)	1.5 (0.5)	< 0.001
SBR Putamen (ant.) (I)	1.9 (0.6)	1.1 (0.5)	2.3 (0.6)	1.5 (0.6)	1.6 (0.7)	< 0.001
SBR Putamen (ant.) (C)	2.1 (0.6)	1.3 (0.5)	2.2 (0.5)	1.6 (0.6)	1.8 (0.7)	< 0.001
SBR Putamen (post.) (I)	1.3 (0.5)	0.7 (0.4)	1.7 (0.4)	1.0 (0.4)	0.9 (0.4)	< 0.001
SBR Putamen (post.) (C)	1.6 (0.5)	0.9 (0.4)	1.8 (0.4)	1.1 (0.5)	1.1 (0.5)	< 0.001
SBR Caudate (I)	1.2 (0.5)	0.9 (0.4)	1.7 (0.5)	1.0 (0.4)	1.2 (0.5)	< 0.001
SBR Caudate (C)	1.4 (0.4)	1.0 (0.5)	1.7 (0.5)	1.1 (0.5)	1.3 (0.5)	< 0.001
SBR Cognitive (I)	1.5 (0.5)	1.0 (0.4)	2.0 (0.5)	1.2 (0.5)	1.4 (0.6)	< 0.001
SBR Cognitive (C)	1.7 (0.5)	1.1 (0.5)	1.9 (0.5)	1.3 (0.5)	1.5 (0.6)	< 0.001
SBR Executive (I)	1.5 (0.5)	1.0 (0.4)	2.0 (0.6)	1.2 (0.5)	1.4 (0.6)	< 0.001
SBR Executive (C)	1.7 (0.5)	1.1 (0.5)	2.0 (0.5)	1.3 (0.6)	1.6 (0.6)	< 0.001
SBR Limbic (I)	1.5 (0.5)	1.0 (0.4)	1.9 (0.5)	1.3 (0.5)	1.4 (0.5)	< 0.001
SBR Limbic (C)	1.7 (0.5)	1.1 (0.4)	1.8 (0.4)	1.4 (0.5)	1.5 (0.6)	< 0.001
SBR Sensorimotor (I)	1.3 (0.5)	0.7 (0.4)	1.7 (0.4)	1.0 (0.4)	1.0 (0.4)	< 0.001
SBR Sensorimotor (C)	1.5 (0.5)	0.9 (0.4)	1.8 (0.4)	1.1 (0.5)	1.2 (0.5)	< 0.001
Cau-Put (ant.) ratio	0.7 [0.5–1.0]	0.8 [0.4–1.0]	0.8 [0.3–0.8]	0.7 [0.2–1.2]	0.8 [0.3–1.9]	< 0.001
Cau-Put (post.) ratio	0.9 [0.5–4.3]	1.2 [0.4–2.0]	1.0 [0.5–1.0]	0.9 [0.4–2.1]	1.3 [0.5–3.2]	< 0.001
AI Ventral Striatum	8.0 [0.8–64.8]	10.2 [0.1–33.9]	6.8 [1.8–21.0]	10.3 [0.1–91.3]	10.7 [0.0–71.9]	0.46
AI Putamen (ant.)	12.6 [0.2–35.2]	15.8 [0.2–82.3]	8.4 [0.2–23.6]	13.1 [0.1–97.5]	14.4 [0.1–107.7]	0.096
AI Putamen (post.)	21.1 [0.7–57.5]	19.7 [0.3–86.7]	5.6 [0.4–16.3]	13.6 [0.0–41.3]	19.7 [0.0–61.6]	< 0.001
AI Caudate	16.7 [0.2–77.1]	16.3 [0.0–142.1]	6.6 [0.6–18.7]	11.7 [0.0–138.3]	13.1 [0.3–92.3]	0.009
Sen-Cog ratio	0.9 [0.2–1.2]	0.8 [0.6–1.5]	0.9 [0.8–1.0]	0.8 [0.5–1.4]	0.7 [0.5–2.2]	< 0.001
Sen-Exe ratio	0.9 [0.3–1.2]	0.7 [0.6–1.7]	0.9 [0.8–1.1]	0.8 [0.5–1.2]	0.7 [0.5–1.8]	< 0.001
Sen-Lim ratio	0.9 [0.2–1.4]	0.8 [0.4–1.2]	1.0 [0.8–1.1]	0.8 [0.4–2.5]	0.7 [0.4–4.6]	< 0.001
AI Cognitive	12.3 [0.1–47.4]	13.7 [0.1–76.7]	4.3 [0.1–9.7]	10.3 [0.1–46.9]	12.6 [0.2–74.4]	0.005
AI Executive	13.0 [0.1–46.4]	15.7 [0.3–106.9]	4.9 [0.3–15.2]	11.4 [0.0–70.2]	13.7 [0.1–85.9]	0.013
AI Limbic	8.4 [0.1–49.5]	10.7 [0.5–50.7]	3.9 [1.2–9.1]	9.1 [0.1–88.7]	11.2 [0.0–77.7]	0.014
AI Sensorimotor	17.7 [0.2–83.2]	24.6 [0.5–77.6]	6.0 [0.6–16.2]	14.7 [2.5–43.2]	17.6 [0.0–92.7]	0.001

Descriptive values are presented as mean (standard deviation) or median [min–max] for continuous variables, and as number (%) for gender

*AI* asymmetry index *(ant.)* anterior, *(C)* contralateral hemisphere relative to the most affected side, *CBD* corticobasal degeneration, *(I)* ipsilateral hemisphere relative to the most affected side, *MSA-P* multiple system atrophy, parkinsonian type, *MSA-C* multiple system atrophy, cerebellar type, *PD* Parkinson’s disease, *PSP* progressive supranuclear palsy, *SBR* standard binding ratio, *(post.)* posterior

*P*-values for group comparisons were obtained using ANOVA for normally distributed continuous variables and the Kruskal–Wallis test for non-normally distributed variables. The chi-square test was used for gender

*P-values for group comparisons were obtained using ANOVA for normally distributed continuous variables and the Kruskal-Wallis test for non-normally distributed variables. The chi-square test was used for sex

## Group differences and discrimination between APS and PD groups using [¹²³I]FP-CIT SPECT-derived metrics

### CBD vs. PD

**Fig. 1:**
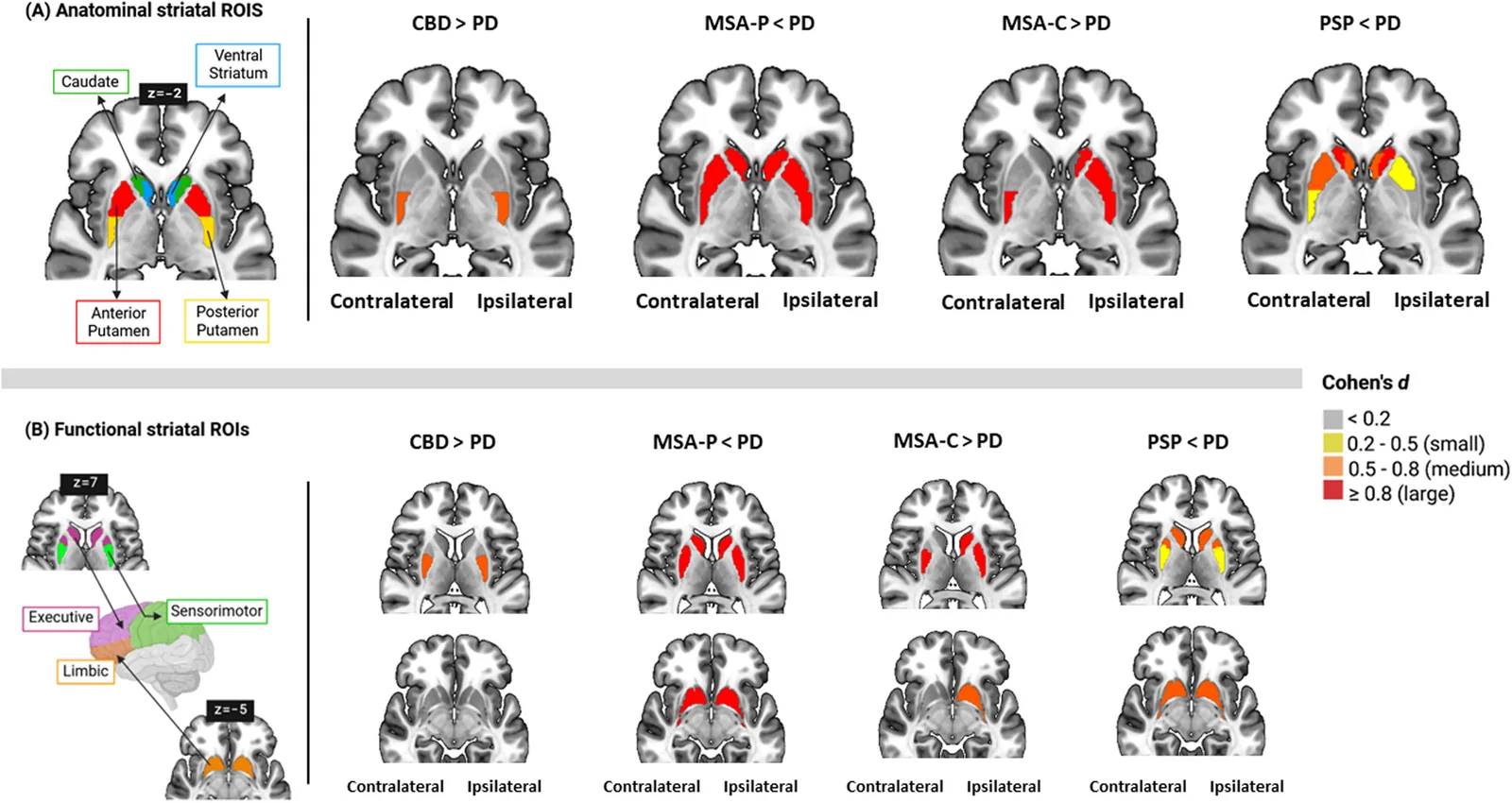
Striatal dopamine transporter binding patterns across atypical parkinsonian syndromes versus Parkinson's disease (PD). **A** Anatomical striatal ROI analysis: Left panel illustrates the anatomical parcellation of the striatum, including the caudate (green), anterior putamen (red), posterior putamen (yellow), and ventral striatum (blue) at MNI coordinate z = 2. Right panels display effect size maps (Cohen’s d) for group differences in [¹²³I]FP-CIT SPECT specific binding ratios (SBR) between each atypical parkinsonian syndrome subtype (CBD, MSA-P, MSA-C, and PSP) and PPD. **B** Functional striatal ROI analysis: Left panel shows the functional subdivision into limbic (orange), executive (magenta), and sensorimotor (light green) regions at MNI coordinates z = 7 and z = −5). Right panels show the corresponding effect size maps for the same group comparisons. Results are presented separately for the ipsilateral and contralateral hemispheres relative to the most affected side. Color scales represent Cohen’s d values, ranging from small (0.2–0.5) to large (> 0.8) effects. Abbreviations: CBD = corticobasal degeneration; MNI = Montreal Neurological Institute; MSA-P = multiple system atrophy, parkinsonian type; MSA-C = multiple system atrophy, cerebellar type; PSP = progressive supranuclear palsy; SBR = specific binding ratio

**Fig. 2:**
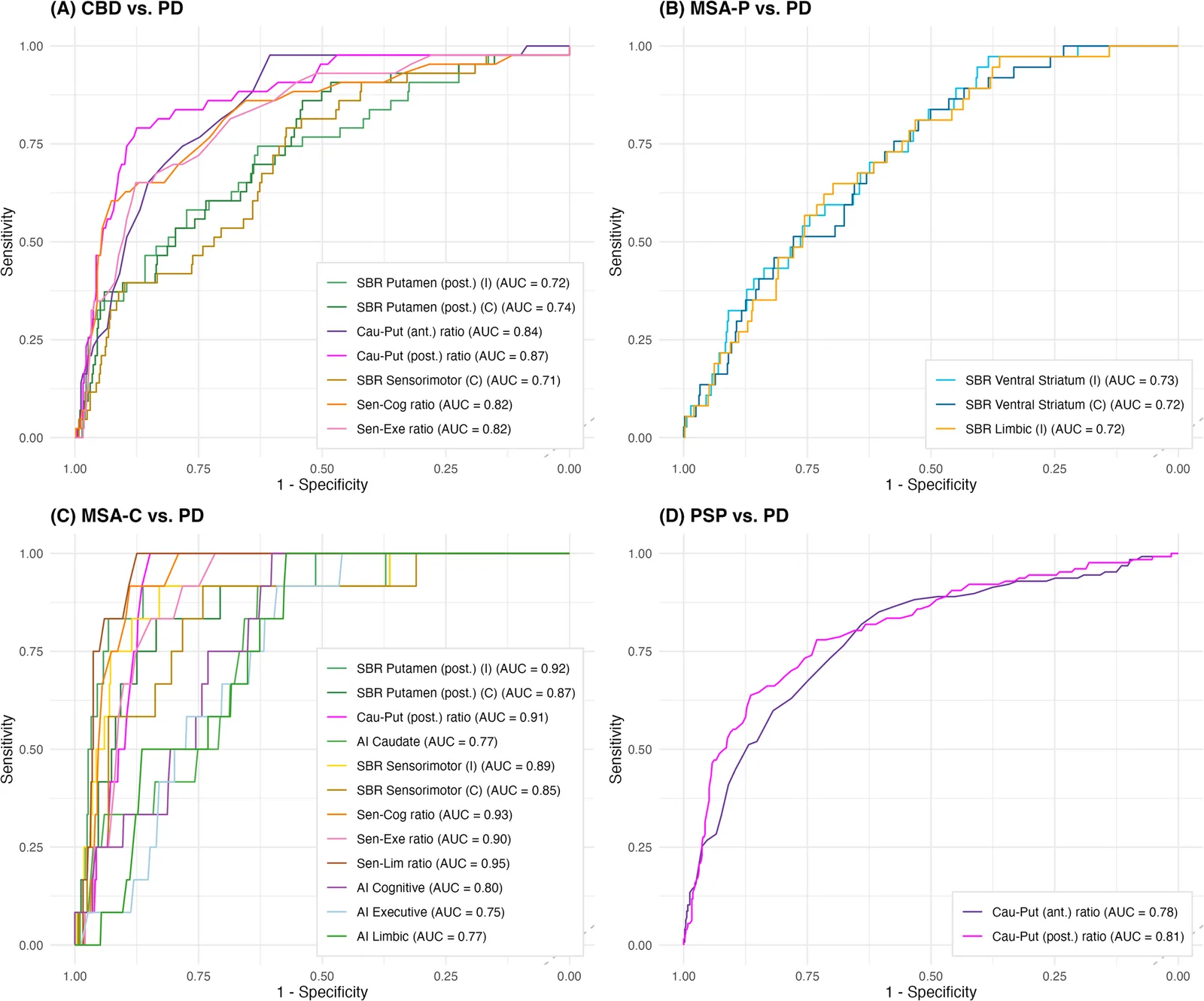
Performance of [¹²³I]FP-CIT SPECT metrics in discriminating atypical parkinsonian syndromes against Parkinson’s disease. Graphs show receiver-operating characteristic (ROC) curves of those significant [¹²³I]FP-CIT SPECT metrics differentiating Parkinson’s Disease (PD) from atypical parkinsonian syndromes: (**A**) CBD, (**B**) MSA-P, (**C**) MSA-C, and (**D**) PSP. Each curve represents a specific anatomical or functional ROI metric that reached statistical significance in the group comparison. Area under the curve (AUC) values are provided to indicate the discriminative power of each biomarker. Abbreviations: Abbreviations: AI = asymmetry index; (ant.) = anterior; (**C**) = contralateral hemisphere relative to the most affected side; CBD = corticobasal degeneration; (**I**) = ipsilateral hemisphere relative to the most affected side; MSA-P = multiple system atrophy, parkinsonian type; MSA-C = multiple system atrophy, cerebellar type; (post.) = posterior; PSP = progressive supranuclear palsy; SBR = specific binding ratio

Compared to the PD group, patients with CBD showed significantly higher [¹²³I]FP-CIT uptake in the bilateral posterior putamen for anatomically defined striatal ROIs (Fig. 1A) and in the bilateral sensorimotor regions for connectivity-based striatal ROIs (Fig. 1B). Furthermore, both the caudate-to-putamen (ant.) and caudate-to-putamen (post.) ratios were lower in the CBD group than in the PD group (Table 2). ROC analyses confirmed that the caudate-to-putamen ratios provided excellent ability to discriminate CBD and PD patients (AUC = 0.84 for caudate-to-putamen (ant.) ratio and AUC = 0.87 for caudate-to-putamen (post.) ratio; Fig. 2A) followed by the sensorimotor-to-cognitive ratio, which also reached a high discriminant AUC (AUC = 0.82; Fig. 2A).

**Table 2:** Pair-wise comparisons of regional FP-CIT SPECT metrics for atypical parkinsonian syndromes against Parkinson’s disease

	CBD vs. PD			MSA-*P* vs. PD			MSA-C vs. PD			PSP vs. PD		
	β (CI)	*p*	d	β (CI)	*p*	d	β (CI)	*p*	d	β (CI)	*p*	d
*Anatomical striatal ROIs*												
SBR Ventral Striatum (I)	−0.1 (−0.2–0.1)	0.305	0.2	−0.5 (−0.6–−0.3)	**< 0.001**	**1**	0.3 (0–0.6)	0.123	0.5	0.3 (0.2–0.3)	**< 0.001**	**0.5**
SBR Ventral Striatum (C)	−0.1 (−0.2–0.1)	0.353	0.2	−0.5 (−0.7–−0.3)	**< 0.001**	**1**	0.1 (−0.2–0.4)	0.577	0.2	0.3 (0.2–0.4)	**< 0.001**	**0.6**
SBR Putamen (ant.) (I)	0.1 (−0.1–0.3)	0.226	0.2	−0.6 (−0.8–−0.4)	**< 0.001**	**1**	0.6 (0.2–0.9)	**0.001**	**0.9**	0.3 (0.1–0.4)	**< 0.001**	**0.4**
SBR Putamen (ant.) (C)	0.1 (−0.1–0.3)	0.19	0.2	−0.7 (−0.9–−0.5)	**< 0.001**	**1.1**	0.3 (0–0.6)	0.058	0.5	0.3 (0.2–0.5)	**< 0.001**	**0.5**
SBR Putamen (post.) (I)	0.3 (0.1–0.4)	**< 0.001**	**0.7**	−0.3 (−0.4–−0.2)	**< 0.001**	**0.8**	0.7 (0.5–0.9)	**< 0.001**	**1.8**	0 (−0.1–0.1)	0.634	0.1
SBR Putamen (post.) (C)	0.3 (0.2–0.5)	**< 0.001**	**0.7**	−0.3 (−0.5–−0.2)	**< 0.001**	**0.8**	0.6 (0.3–0.8)	**< 0.001**	**1.3**	0.1 (0–0.2)	**0.048**	**0.2**
SBR Caudate (I)	−0.1 (−0.3–0)	0.065	0.3	−0.4 (−0.6–−0.3)	**< 0.001**	**1**	0.3 (0–0.6)	**0.038**	**0.8**	0.3 (0.3–0.4)	**< 0.001**	**0.8**
SBR Caudate (C)	−0.1 (−0.2–0)	0.191	0.2	−0.4 (−0.6–−0.3)	**< 0.001**	**1**	0.2 (−0.1–0.5)	0.191	0.4	0.4 (0.3–0.5)	**< 0.001**	**0.8**
Cau-Put (ant.) ratio	0.8 (0.8–0.9)	**< 0.001**	**1**	1 (0.9–1.1)	0.987	0	0.9 (0.8–1)	0.185	0.4	1.2 (1.1–1.2)	**< 0.001**	**0.8**
Cau-Put (post.) ratio	0.8 (0.7–0.8)	**< 0.001**	**1**	0.9 (0.9–1)	0.164	0.2	0.7 (0.6–0.8)	**< 0.001**	**1.2**	1.3 (1.2–1.4)	**< 0.001**	**1**
AI Ventral Striatum	1.1 (0.8–1.4)	0.674	0.1	1 (0.7–1.3)	0.924	0	0.7 (0.4–1.1)	0.294	0.4	0.8 (0.7–1)	0.171	0.2
AI Putamen (ant.)	0.8 (0.6–1)	0.099	0.3	1.1 (0.8–1.5)	0.499	0.1	0.5 (0.3–0.9)	0.062	0.7	1.2 (1–1.4)	0.152	0.2
AI Putamen (post.)	1 (0.8–1.2)	0.865	0	1.3 (1–1.6)	0.051	0.4	0.3 (0.2–0.5)	**< 0.001**	**1.7**	1.4 (1.2–1.6)	**< 0.001**	**0.5**
AI Caudate	1.3 (1–1.7)	0.075	0.3	1.6 (1.2–2.1)	**0.004**	**0.5**	0.4 (0.3–0.7)	**0.002**	**1**	0.9 (0.8–1.1)	0.194	0.1
*Functional striatal ROIs*												
SBR Cognitive (I)	0 (−0.2–0.2)	0.999	0	−0.5 (−0.7–−0.4)	**< 0.001**	**1**	0.4 (0.1–0.8)	**0.007**	**0.9**	0.3 (0.2–0.4)	**< 0.001**	**0.5**
SBR Cognitive (C)	0 (−0.1–0.2)	0.831	0	−0.6 (−0.7–−0.4)	**< 0.001**	**1**	0.2 (−0.1–0.5)	0.143	0.5	0.4 (0.2–0.5)	**< 0.001**	**0.7**
SBR Executive (I)	0 (−0.2–0.2)	0.99	0	−0.5 (−0.7–−0.4)	**< 0.001**	**1**	0.5 (0.2–0.8)	**0.005**	**0.9**	0.3 (0.2–0.4)	**< 0.001**	**0.6**
SBR Executive (C)	0 (−0.1–0.2)	0.81	0	−0.6 (−0.7–−0.4)	**< 0.001**	**1**	0.3 (0–0.6)	0.093	0.5	0.4 (0.3–0.5)	**< 0.001**	**0.7**
SBR Limbic (I)	0 (−0.2–0.2)	0.975	0	−0.5 (−0.7–−0.4)	**< 0.001**	**1**	0.3 (0.1–0.6)	**0.02**	**0.7**	0.2 (0.1–0.3)	**< 0.001**	**0.5**
SBR Limbic (C)	0 (−0.1–0.2)	0.889	0	−0.5 (−0.7–−0.4)	**< 0.001**	**1**	0.1 (−0.1–0.4)	0.403	0.3	0.3 (0.2–0.4)	**< 0.001**	**0.6**
SBR Sensorimotor (I)	0.2 (0.1–0.4)	**0.009**	**0.5**	−0.4 (−0.5–−0.2)	**< 0.001**	**0.9**	0.6 (0.4–0.8)	**< 0.001**	**1.5**	0.1 (0–0.2)	**0.019**	**0.3**
SBR Sensorimotor (C)	0.2 (0.1–0.4)	**0.001**	**0.5**	−0.4 (−0.5–−0.2)	**< 0.001**	**0.8**	0.5 (0.3–0.8)	**< 0.001**	**1.2**	0.2 (0.1–0.3)	**0.001**	**0.4**
Sen-Cog ratio	1.2 (1.1–1.2)	**< 0.001**	**1**	1 (1–1.1)	0.105	0.3	1.2 (1.1–1.4)	**< 0.001**	**1.2**	0.9 (0.9–0.9)	**< 0.001**	**0.6**
Sen-Exe ratio	1.2 (1.1–1.2)	**< 0.001**	**1**	1.1 (1–1.1)	**0.019**	**0.4**	1.2 (1.1–1.3)	**< 0.001**	**1.1**	0.9 (0.9–0.9)	**< 0.001**	**0.8**
Sen-Lim ratio	1.2 (1.1–1.3)	**< 0.001**	**0.6**	1 (0.9–1.1)	0.84	0	1.3 (1.1–1.5)	**0.004**	**0.9**	0.9 (0.9–1)	**0.014**	**0.3**
AI Cognitive	1 (0.8–1.3)	0.967	0	1.3 (1–1.7)	0.131	0.3	0.3 (0.2–0.5)	**< 0.001**	**1.4**	1.1 (1–1.3)	0.131	0.2
AI Executive	1 (0.8–1.3)	0.855	0	1.3 (1–1.8)	0.078	0.4	0.4 (0.2–0.6)	**< 0.001**	**1.1**	1.1 (1–1.3)	0.212	0.1
AI Limbic	1 (0.7–1.3)	0.892	0	1.2 (0.9–1.6)	0.551	0.2	0.4 (0.2–0.6)	**< 0.001**	**1.1**	1 (0.9–1.2)	0.892	0
AI Sensorimotor	1.1 (0.9–1.4)	0.501	0.1	1.4 (1.1–1.8)	0.007	0.5	0.4 (0.3–0.6)	**< 0.001**	**1.3**	1.2 (1–1.3)	0.061	0.2

Values represent standardized regression coefficients (β) with 95% confidence intervals (CI), *p*-values, and Cohen’s *d* effect sizes for pairwise group comparisons of FP-CIT specific binding ratios (SBRs), ratios, and asymmetry indices (AI) in anatomical and functional striatal regions of interest (ROIs). Comparisons were adjusted for age, sex, and disease duration. For SBR metrics, β denotes the absolute mean difference between each APS group and the PD group, adjusted for age, sex, and disease duration. For ratio and asymmetry index (AI) variables, generalized linear models with a Gamma distribution and log-link function were fitted. In these cases, the reported β indicates the ratio of means, defined as the mean value in the APS group divided by the mean value in the PD group. Values less than 1 indicate lower values in the APS group compared to PD, values greater than 1 indicate higher values, and values near 1 suggest no meaningful difference. Confidence intervals are reported on the same scale. Bold typeface highlights statistically significant pair-wise comparisons (p < 0.05)

*AI* asymmetry index; *(ant.)* anterior, *(C)* contralateral hemisphere relative to the most affected side, *CBD* corticobasal degeneration, *(I)* ipsilateral hemisphere relative to the most affected side, *MSA-P* multiple system atrophy, parkinsonian type, *MSA-C* multiple system atrophy, cerebellar type, *PD* Parkinson’s disease, *PSP* progressive supranuclear palsy, *SBR* standard binding ratio, *(post)* posterior

### MSA-P vs. PD

Compared to PD, the MSA-P group had a significantly reduced global striatal [¹²³I]FP-CIT uptake compared to PD, (Fig. 1A and B). However, regional uptake metrics showed poor discriminative capacity overall. Ventral striatum SBRs reached a moderate discriminative ability with an AUC of 0.73 and 0.72 for the ipsilateral and contralateral sides, respectively, as well as limbic ipsilateral region with AUC of 0.72 (Fig. 2B).

### MSA-C vs. PD

The MSA-C group showed significantly higher [¹²³I]FP-CIT SBR values compared to PD in the bilateral posterior putamen using anatomically defined ROIs (Fig. 1A) and in the sensorimotor region using connectivity-defined ROIs (Fig. 1B). ROC analyses further demonstrated strong discriminative power for the caudate-to-putamen (post.) ratio achieving an AUC of 0.91 and for the sensorimotor-to-limbic ratio with an AUC of 0.95 (Fig. 2C). Due to the small sample size of the MSA-C group (*n* = 12), sensitivity and LR− values calculated at the Youden-optimal threshold should be interpreted with caution. These estimates are derived from a limited number of cases and may be less stable and generalizable than the AUC measures. For transparency and to support independent verification, the corresponding true positive (TP), true negative (TN), false positive (FP), and false negative (FN) counts are provided in Supplementary Table S1.

### PSP vs. PD

The PSP group showed significantly reduced striatal [¹²³I]FP-CIT binding across all ROIs with respect to PD (Fig. 1A and B). Additionally, a lower caudate-to-putamen ratios were reported in PSP compared to PD (Table 2), which showed moderate to high discriminatory power (AUC = 0.78 for caudate-to-putamen (ant.) and AUC = 0.81 for caudate-to-putamen (post.); Fig. 2D). No other metric reached a fair discriminatory capacity for distinguishing between the two groups (see Supplementary Table S1).

### Group differences and discrimination across APS groups using [¹²³I]FP-CIT SPECT-derived metrics

Overall, results across pairwise comparisons revealed that SBR values in the posterior putamen and sensorimotor ROIs consistently emerged as the most reliable discriminators between the different APS groups, with caudate-to-putamen ratios providing complementary diagnostic information (Supplementary Tables S2–S3). The CBD group had higher uptake than MSA-P and PSP, particularly in putamen and sensorimotor regions, with posterior putamen metrics showing the best discriminatory power (AUC ≈ 0.83–0.85 for CBD vs. MSA-P; AUC ≈ 0.68–0.77 for CBD vs. PSP). Functional and anatomical parcellation uncovered interhemispheric asymmetries between CBD and MSA-C. The sensorimotor asymmetry index demonstrated moderate discriminative performance (AUC = 0.79), whereas the posterior putamen asymmetry index achieved the highest discrimination between the two groups (AUC = 0.83). In MSA subtypes, MSA-P exhibited consistently lower uptake than MSA-C across all striatal regions anatomically, with posterior putamen metrics achieving excellent accuracy (AUC ≈ 0.93–0.96), while functional parcellation confirmed these findings with sensorimotor ROIs as the strongest discriminators. PSP showed lower FP-CIT uptake than MSA-C in both anatomical and connectivity-based ROIs, with posterior putamen and sensorimotor indices providing high discrimination (AUC ≈ 0.88–0.89), whereas comparisons with MSA-P revealed only a lower caudate-to-putamen ratios in PSP, which reached moderate accuracy (AUC ≈ 0.73–0.74).

### Diagnostic classification model

The Random Forest model, trained on disease duration-adjusted [¹²³I]FP-CIT SPECT metrics, achieved an overall accuracy of 0.64 across 10 × 10 repeated stratified cross-validation. Given the imbalanced multiclass nature of the dataset, overall accuracy is not the most informative primary performance metric. Macro-balanced accuracy, macro F1, and per-class metrics offer a more comprehensive assessment of model performance. The model achieved a macro balanced accuracy of 0.687 and a macro F1 score of 0.432 (Table 3A). Detailed per-class performance is presented in Table 3B. Parkinson’s disease (PD) and multiple system atrophy-cerebellar type (MSA-C) demonstrated the highest balanced accuracy (0.73 and 0.72, respectively). PD exhibited the highest sensitivity (0.73) and precision (0.86). MSA-C showed the highest specificity (0.98), indicating a low false-positive rate; however, sensitivity and precision were modest, so predictions should be interpreted as high-confidence flags rather than reliable case detection. Progressive supranuclear palsy (PSP) showed intermediate performance across metrics. Corticobasal degeneration (CBD) and multiple system atrophy-parkinsonian type (MSA-P) exhibited lower sensitivity and precision, but maintained high specificity (> 0.92). F1 scores reflected these trends, ranging from 0.79 for PD to 0.27 for MSA-P.

**Table 3:** Multiclass classification performance of the Random Forest model based on disease duration-adjusted [¹²³I]FP-CIT SPECT metrics

(A) Overall statistics					
Accuracy = 0.64 ± 0.004^*^					
Macro Balanced Accuracy = 0.68					
Macro Sensitivity = 0.48					
Macro Precision = 0.41					
Macro F1 = 0.43					

(B) Random Forest classification performance by diagnostic group					
	**CBD**	**MSA-C**	**MSA-P**	**PSP**	**PD**
n	43	12	37	127	487
Balanced Accuracy	0.63	0.72	0.65	0.68	0.73
Sensitivity	0.32	0.45	0.37	0.51	0.73
Specificity	0.94	0.98	0.92	0.84	0.74
Precision (PPV)	0.24	0.30	0.21	0.42	0.86
F1	0.28	0.36	0.27	0.46	0.79

Random Forest performance metrics computed on accumulated out-of-fold predictions across 100 cross-validation iterations

*CBD* corticobasal degeneration, *MSA-P* multiple system atrophy, parkinsonian type, *MSA-C* multiple system atrophy, cerebellar type, *PD* Parkinson’s disease, *PSP* progressive supranuclear palsy

^*^A naive majority-class classifier that always predicts PD would achieve an overall accuracy of 0.69 (487/706), but would yield zero sensitivity for all APS groups. Consequently, overall accuracy must be interpreted in the context of class imbalance. Macro balanced accuracy, macro F1, and per-diagnostic group metrics provide more informative measures of diagnostic utility in this cohort

**Fig. 3:**
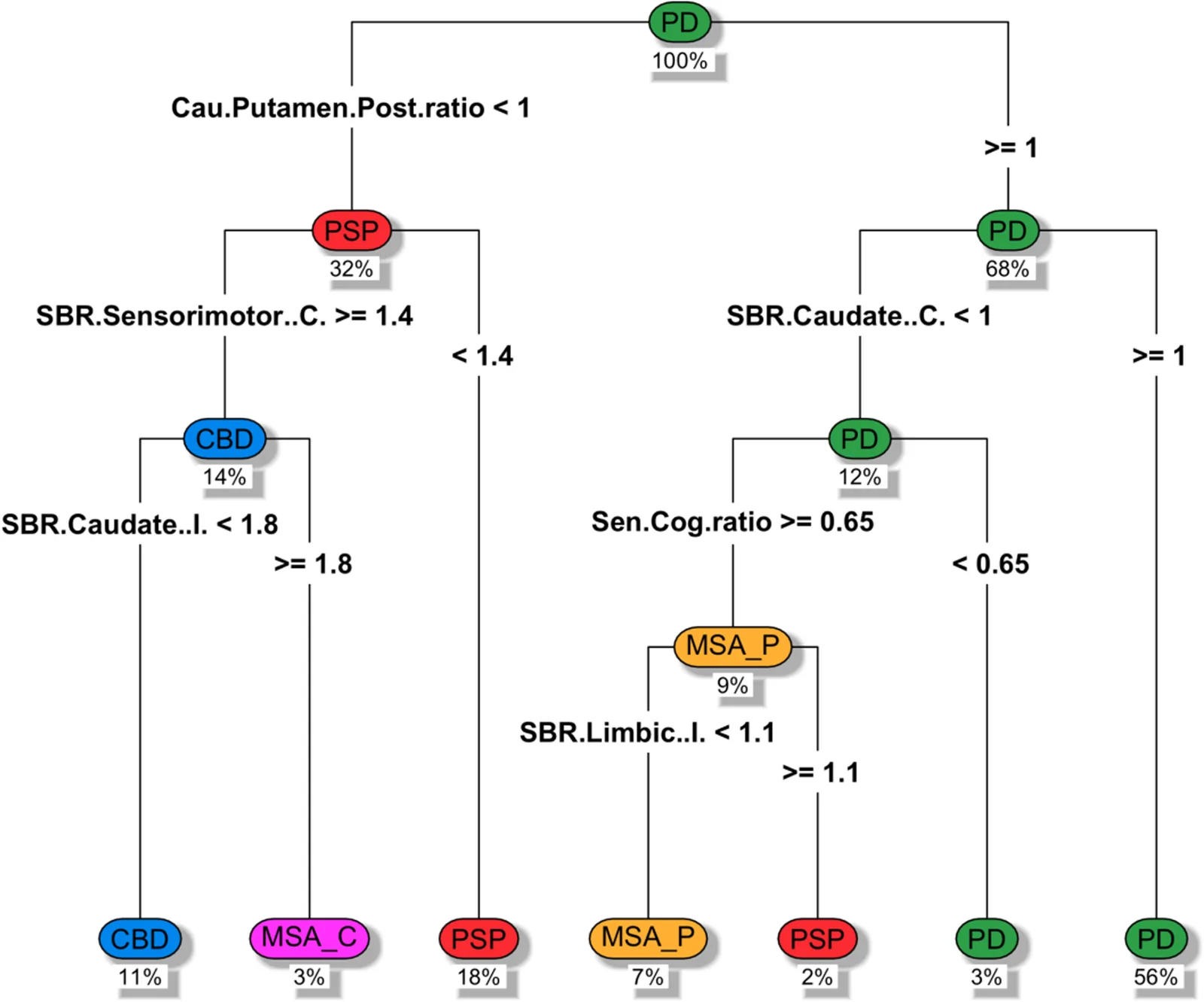
Multiclass Random Forest classification analysis for the differential diagnosis of degenerative parkinsonian syndromes. Distilled decision tree summarising the main classification pathways of the Random Forest model trained on disease duration-residualized [¹²³I]FP-CIT SPECT semiquantitative metrics. The tree was fitted to the out-of-fold Random Forest predicted labels (fidelity = 81%) using the one-standard-error rule for complexity parameter selection (cp = 0.015) and a maximum depth of 4 nodes. Each node shows the predicted diagnostic class and the proportion of out-of-fold observations at that node. Thresholds are expressed in original [¹²³I]FP-CIT SPECT metric units, back-transformed at the median disease duration of the cohort (2.6 years) for clinical interpretability. Abbreviations: AI = asymmetry index; (ant.) = anterior; (**C**) = contralateral hemisphere relative to the most affected side; CBD = corticobasal degeneration; (**I**) = ipsilateral hemisphere relative to the most affected side; MSA-P = multiple system atrophy, parkinsonian type; MSA-C = multiple system atrophy, cerebellar type; (post.) = posterior; PSP = progressive supranuclear palsy; SBR = specific binding ratio

Figure 3 presents a distilled decision tree that offers a simplified visualization of the classification patterns identified by the Random Forest model. As it is fitted to the out-of-fold Random Forest predictions, the tree reproduces the ensemble’s decisions with 81% fidelity. As shown in this decision-tree, the posterior caudate-to-putamen ratio is identified as the primary split: cases with lower values (< 1) were predominantly classified as PSP, whereas those with higher values entered a second branch further split by the [¹²³I]FP-CIT of the contralateral caudate. Within this higher-ratio branch, lower caudate [¹²³I]FP-CIT SBR values triggered additional splits based on the sensorimotor-to-cognitive ratio and the further stratification by the ipsilateral caudate SBR separating CBD from MSA-C. Cases not meeting these criteria were classified as PSP at intermediate nodes. Supplementary Figure S1 illustrates the ranking of [¹²³I]FP-CIT SPECT metrics by their contribution to classification, averaged across all 100 cross-validation folds (based on the mean decrease in Gini impurity). The caudate-to- posterior and anterior putamen and the asymmetry index of the posterior putamen emerged as the most informative variables, followed by the sensorimotor-to-limbic and sensorimotor-to-executive ratios.

## Discussion

This study demonstrates that [¹²³I]FP-CIT SPECT-derived semi-quantitative metrics, when analyzed using anatomical and functional striatal parcellations, can improve differential diagnosis between PD and APS, including PSP, MSA-P, MSA-C, and CBD. Our findings show that integrating these quantitative indices within a machine learning framework enhances diagnostic accuracy supporting traditional visual or ROI-based assessments. Of the metrics evaluated, the caudate-to-putamen ratio, the sensorimotor-to-limbic ratio and regional asymmetry indices were the most consistent predictors for distinguishing PD from APS, whereas putaminal and sensorimotor regional uptake, with their asymmetry indices, provided the best discrimination between different APS subtypes. Notably, combining quantification within anatomical and functional striatal parcellations revealed disease-specific patterns of dopaminergic loss that were not apparent using conventional methods, highlighting the clinical potential of [¹²³I]FP-CIT SPECT and data-driven models in classifying parkinsonian disorders.

Our findings confirm the well-established pattern of striatal dopaminergic loss associated with PD. Semiquantitative [¹²³I]FP-CIT SPECT analysis revealed reduced striatal uptake with an anteroposterior gradient, with the most pronounced decrease observed in the posterior putamen and the sensorimotor functional region. This likely reflects the known neuropathological basis of PD, involving predominant degeneration of the ventrolateral region of the substantia nigra pars compacta (pcSN) that projects to the posterior putamen, while the caudate is relatively spared. There is also a higher α-synuclein burden in this nigral region, which is inversely proportional to DaT density [36, 37]. Consistently, greater involvement of the putamen compared to the caudate and of the sensorimotor region compared to the cognitive region supports the association between striatal DaT deficiency on SPECT imaging and PD severity and motor involvement [38], demonstrating the anatomical-functional correspondence of the putaminal region.

When comparing PD and APS, two semi-quantitative ratios consistently emerged as the most reliable discriminative metrics. The caudate-to-putamen (post.) ratio, derived from anatomical parcellation, robustly differentiated PD from CBD, PSP and MSA-C. Meanwhile, the sensorimotor-to-cognitive ratio, obtained from functional striatal subdivisions, proved particularly useful for distinguishing PD from CBD and MSA-C. These findings are consistent with and extend previous reports indicating that the putaminal signal is more commonly used for differential diagnosis of parkinsonian syndromes and caudate-to-putamen ratio is an informative [¹²³I]FP-CIT SPECT indices relatively unaffected by background activity or reconstruction parameters [13, 16]. Additionally, we analyzed [¹²³I]FP-CIT uptake across anatomically and functionally defined striatal subregions to characterize topographic distribution patterns specific to each APS.

In patients with CBD, the functional parcellation revealed relatively lower [¹²³I]FP-CIT uptake in the sensorimotor subregion compared with other striatal territories. This finding aligns with the characteristic neuropathological pattern of CBD, which predominantly involves posterior cortical regions, such as the parietal lobe and posterior frontal cortex, that are functionally connected to the striatal sensorimotor subregion [20]. However, when compared with the other parkinsonian syndromes, CBD showed significantly higher [¹²³I]FP-CIT uptake across both anatomical and functional striatal regions than PD, PSP and MSA-P, particularly in the posterior putamen and sensorimotor territories, which allow robust group differentiation. Moreover, in line with the typically asymmetric clinical presentation of CBD as reflected in established diagnostic criteria [27], our data showed particularly pronounced posterior putaminal asymmetry in CBD relative to these parkinsonian syndromes. Accordingly, asymmetry indices in this subregion were highest in CBD, closely followed by PD and MSA-P with comparable values, whereas PSP showed a more symmetric pattern than these groups. By contrast, MSA-C exhibited markedly lower striatal asymmetry, and discrimination between CBD and MSA-C was driven mainly by lateralization indices, particularly posterior putamen and sensorimotor regions (AUC up to 0.832), reflecting the marked hemispheric predominance of dopaminergic dysfunction in CBD versus the more symmetric striatal involvement in MSA-C [39]. Our findings indicate that both CBD and MSA-C exhibit relatively less pronounced nigrostriatal degeneration compared to the other parkinsonian syndromes, yet show distinct topographic patterns of dopaminergic loss, highlighting the value of lateralization metrics for differentiating these two APS subtypes.

In PSP, striatal parcellations revealed predominant involvement of the caudate and posterior putamen, with relative preservation of the anterior putamen and ventral striatum, consistent with the ventrodorsal gradient previously described in this syndrome [13, 40]. Clinically, this topography is plausible because caudate involvement likely reflects disruption of frontostriatal circuits underlying the executive and behavioral features of PSP, whereas posterior putaminal involvement is consistent with the degeneration of the nigrostriatal pathway underlying the parkinsonian motor syndrome [41]. Compared with PD and MSA-P, PSP showed diffuse and symmetrical reductions in [¹²³I]FP-CIT uptake across the striatum, and accordingly the caudate-to-putamen (post.) ratio provided the best group discrimination. Finally, PSP differed most clearly from MSA-C, with discrimination driven by lower uptake in PSP across the posterior putamen, caudate, sensorimotor and cognitive territories, particularly within the executive subregion, consistent with greater frontostriatal circuit involvement underlying the more fronto-subcortical phenotype and more pronounced nigrostriatal degeneration that characterize PSP, whereas MSA-C is more strongly dominated by disautonomic and cerebellar features [24, 41]. In MSA, we analyzed the parkinsonian (MSA-P) and cerebellar (MSA-C) subtypes separately, given their recognized clinical and pathological differences [24]. Compared with other APS, patients with MSA-C showed a mild and relatively symmetric reduction in [¹²³I]FP-CIT uptake across all striatal subregions. In our cohort 16.7% of studies were reported as within normal limits while 50% showed only mild reduction. By contrast, MSA-P exhibited the most severe dopaminergic loss, especially in the ipsilateral posterior putamen. Consequently, the posterior putamen and sensorimotor territories provided an effective means of discriminating between the two subtypes, with higher striatal ratios and asymmetry indices in MSA-P indicating more pronounced interhemispheric differences. Overall, these observations are consistent with previous reports showing that MSA-C often presents with borderline or even normal uptake values, and with low caudate-to-putamen ratios and asymmetry indices [42, 43]. The observed imaging differences in this study reflect distinct neuropathological substrates: striatonigral degeneration predominates in MSA-P, whereas olivopontocerebellar pathology is the leading process in MSA-C [44]. From a clinical standpoint, it is essential to distinguish these phenotypes during SPECT interpretation, since [¹²³I]FP-CIT imaging is most informative in MSA-P and less sensitive in MSA-C, where scans may appear normal at disease onset [39, 45]. However, a normal SPECT scan does not rule out MSA-C, so follow-up imaging with standardized quantification is recommended if there is still clinical suspicion [46, 47]. Related to PD, MSA-C showed relatively higher and more symmetric DAT binding particularly in the posterior putamen and bilateral sensorimotor regions, allowing clear discrimination between groups. In this comparison, both the caudate-to-putamen (post.) ratio (AUC = 0.91) and the sensorimotor-to-limbic ratio (AUC = 0.95) demonstrated excellent diagnostic accuracy, while the posterior putamen asymmetry index also showed good discrimination (AUC = 0.82), supporting the added value of functional parcellation metrics and asymmetric metrics for identifying the MSA-C phenotype. Given the small MSA-C sample (*n* = 12), the apparent perfect sensitivity (sensitivity = 1.000; LR− = 0.00) may produce unstable point estimates and should not be interpreted as perfect rule-out performance.

When we compared MSA-P with PD, MSA-P exhibited a more severe and diffuse reduction in striatal binding, particularly in the putamen. However, the striatal patterns on [¹²³I]FP-CIT SPECT were broadly similar between the two conditions, with substantial overlap that may limit reliable differentiation. This is consistent with the pattern of presynaptic dopaminergic dysfunction characteristic of α-synucleinopathies, reflecting their shared neuropathological substrate [13, 39, 42, 44, 48]. In our cohort, regional indices demonstrated only modest diagnostic performance, in the ventral striatum (AUC = 0.73) and, within the cognitive area, the ipsilateral limbic subregion (AUC = 0.72). The relatively low AUC for PD versus MSA-P, is consistent with the well-recognized difficulty of this differential diagnosis and likely reflects substantial overlap in presynaptic dopaminergic imaging patterns between both disorders. This finding supports the need for multimodal approaches beyond semiquantitative [¹²³I]FP-CIT SPECT alone. Complementary neuroimaging modalities, such as cardiac ¹²³I-MIBG scintigraphy or ¹⁸F-FDG PET, may enhance differential diagnosis by detecting changes beyond nigrostriatal degeneration, such as differences in cardiac sympathetic denervation or regional cortical hypometabolism [44, 47]. In summary, our results demonstrate that, when analyzed using both anatomical and functional striatal parcellations, [¹²³I]FP-CIT SPECT derived semiquantitative metrics can enhance differential diagnosis between PD and APS subtypes. To further improve diagnostic accuracy, we integrated these quantitative indices into a machine learning framework and developed a random forest classification model. Previous studies have identified subregional patterns of preferential striatal dopamine transporter loss that may aid the differentiation of degenerative parkinsonian syndromes [13, 16], while other studies have applied support-vector-machine (SVM) models to distinguish PD from vascular parkinsonism [12] and to classify degenerative parkinsonian syndromes using conventional anatomical striatal indices derived from [¹²³I]FP-CIT SPECT [17]. In contrast, the present study extends prior work by integrating functional connectivity-based striatal ROIs together with conventional anatomical metrics within a multiclass Random Forest framework, including PD, PSP, MSA-C, MSA-P, and CBD. This approach enables simultaneous multiclass classification across all diagnostic groups within a single framework, naturally captures non-linear relationships and feature interactions among striatal metrics, and provides variable importance measures that facilitate biological interpretation of the classification results [34].

As illustrated in Fig. 3, the caudate-to-posterior putamen ratio emerged as the main determinant of classification, defining two dominant diagnostic pathways. Lower ratios group PSP, CBD and MSA-C cases. PSP is characterized by lower contralateral sensorimotor uptakes, while higher uptake and further splits based on ipsilateral caudate binding refined differentiation between CBD and MSA-C, capturing the more preserved overall uptake in MSA-C with respect to CBD and PSP. Conversely, higher caudate-to-posterior putamen ratio grouped PD and MSA-P, where reduced anterior striatal binding (lower contralateral caudate uptake and higher sensorimotor-to-cognitive ratio) was indicative of MSA-P, while higher contralateral caudate uptake favored PD. A minority of PSP cases were grouped in this branch, sharing a similar binding behavior than MSA-P but with lower ipsilateral limbic uptake.

Despite the strong overall performance, a 14% PD misclassification rate suggests a clinically relevant subgroup with overlapping [¹²³I]FP-CIT SPECT patterns, probably with PSP or MSA-P [6]. This highlights the persistent difficulty of PD–APS differentiation and supports combining DaT-SPECT with multimodal biomarkers and longitudinal follow up in borderline cases. In this context, it is noteworthy that in PSP, traditional metrics such as putaminal SBR alone are often insufficient for accurate discrimination [17], highlighting the importance of ratio-based measures and machine learning approaches to improve differentiation from PD. The ROC analysis and variable importance in the diagnostic classification model in our study confirmed these as the most influential predictors, consistent with known striatal dopaminergic gradients: the caudate-to-putamen ratios reflecting the posterior-to-anterior gradient of PD, and the sensorimotor-to-limbic ratio capturing the relative sparing of limbic regions in MSA-C. Although asymmetry metrics alone did not reliably distinguish PD from APS, their integration with regional uptake ratios in a machine learning framework improved subtype differentiation. Therefore, the variable hierarchy established by the classification model emphasizes the importance of asymmetry ratios and indices over traditional uptake metrics (SBRs) when addressing diagnostic differentiation of parkinsonian syndromes, shifting the classic paradigm focused on the evaluation of regional uptake alterations. Overall, the model revealed clear and physiologically coherent decision rules that mirror clinical reasoning, underscoring the translational potential of machine learning–based classification in the diagnostic assessment of parkinsonian syndromes.

A major strength of this study is its large cohort of 487 patients with PD and 219 patients with APS, which enabled robust statistical comparisons across the full spectrum of degenerative parkinsonian syndromes. Using a dual anatomical–functional striatal parcellation framework alongside a standardized [¹²³I]FP-CIT SPECT pipeline ensured that the quantitative metrics were highly reproducible and biologically interpretable. Additionally, applying an interpretable random forest classifier, trained on disease duration-adjusted semiquantitative metrics from anatomical and functional striatal parcellations represents a novel, clinically relevant approach to differential diagnosis, extending our group’s previous machine learning work in parkinsonism. Nevertheless, several limitations should be acknowledged. Diagnoses were based on clinical criteria without pathological confirmation, and the cross-sectional design precluded assessment of diagnostic revisions over time (“switchers”), including whether misclassified cases were enriched among patients with diagnostic change during follow-up. Fluid biomarkers (α-synuclein, tau, NfL) were not systematically available in our cohort, but future multimodal studies could combine them with [¹²³I]FP-CIT SPECT metrics. The laterality criterion (ipsilateral/contralateral) for determining the hemisphere most affected by striatal damage is based exclusively on semiquantification of the posterior putamen. Briefly acknowledge that the primary model uses posterior putamen SBR both for laterality assignment and as a discriminative feature, and that the raw left/right sensitivity analysis was performed to assess the robustness of the findings to this feature-engineering choice (Supplementary Table S8 and Figure S3). Although this methodology is accurate, robust, and reproducible for this purpose, its combined use with clinical assessment is recommended to maximize discrimination between clinical subtypes [22, 49]. However, due to the retrospective design, these clinical data are unavailable for most of the cohort, precluding the benefits of their complementary use. Although asymmetry ratios showed diagnostic value in our cohort, the present study was not designed to establish definitive clinical cut-off values. The significant age difference between diagnostic groups, with PSP and CBD patients being on average approximately 12 years older than PD and MSA patients, may contribute to observed differences in SBR values, given the well-documented age-related decline in nigrostriatal DAT binding. Although age was included as a covariate in all pairwise statistical comparisons, its potential influence on the imaging metrics cannot be entirely excluded, and future studies with better age-matched groups would strengthen causal interpretation. The severe imbalance between the PD and APS groups, as well as the small sample sizes of certain APS subtypes, particularly MSA-C (*n* = 12), constrained per-class classification performance despite the use of SMOTE and repeated cross-validation. The overall accuracy of 64% should be interpreted in the context of this class imbalance, as majority-class dominated metrics do not adequately reflect the model’s discriminative capacity for minority APS groups. Per-class balanced accuracies (0.62–0.73) and high specificities (> 0.84) provide a more informative picture of clinical utility. High specificity reflects a low false-positive rate, but modest sensitivity, particularly for CBD and MSA-P, implies that many true cases may be missed or misclassified; therefore, the model should be interpreted as a high-specificity decision-support aid rather than a screening tool. In terms of clinical positioning, this tool is best conceived as a quantitative decision-support system rather than a standalone classifier, providing an objective ‘second opinion’ to flag cases requiring closer clinical scrutiny. However, although a formal head-to-head comparison with expert visual reads was not feasible within the scope of this study, it represents an important direction for future validation work. Furthermore, [¹²³I]FP-CIT SPECT has lower spatial resolution than PET, which could limit fine-grained striatal delineation. To confirm the generalizability and clinical utility of this approach, external validation in independent datasets, integration of multimodal imaging data, and longitudinal follow-up will be essential next steps.

## Conclusion

In conclusion, this study demonstrates that the differential diagnosis of parkinsonian syndromes is enhanced by the semiquantitative analysis of [¹²³I]FP-CIT SPECT using complementary anatomical and functional striatal parcellations. Integrating these quantitative imaging metrics into an interpretable random forest model provided a robust, biologically coherent classification of PD and APS subtypes, revealing dopaminergic loss patterns that are consistent with known neuropathological gradients. Identifying key indices, such as the caudate-to-putamen and sensorimotor-to-limbic ratios, as well as certain asymmetry indices, highlights their potential as practical biomarkers for in vivo disease differentiation. By combining quantitative neuroimaging and machine learning, this framework promotes the development of data-driven tools to improve diagnostic accuracy and the clinical decision process in movement disorders. Future studies should focus on external validation, longitudinal evaluation and the exploration of multimodal models that integrate dopaminergic imaging with other functional neuroimaging techniques.

## Supplementary Information

Below is the link to the electronic supplementary material.

Supplementary Material 1.

## Data Availability

The datasets generated and analysed during the current study are available from the corresponding author on reasonable and formal request approved by the relevant local ethics committees.
