# Supplementary material for: Semiquantitative [¹²³I]FP-CIT SPECT metrics combined with machine learning improve clinical differentiation of Parkinson’s disease and atypical parkinsonian syndrome
Source: Eur J Nucl Med Mol Imaging. 2026 Jul 3;53(12):7391–406. doi: 10.1007/s00259-026-08022-x (PMC13633342; doi:10.1007/s00259-026-08022-x)
Supplement: Supplementary file 1 [file 259_2026_8022_MOESM1_ESM.docx]

| **Table S1. Diagnostic performance of FP-CIT metrics for distinguishing each atypical parkinsonian syndrome from PD** | | | | | | | | | | | | |
| --- | --- | --- | --- | --- | --- | --- | --- | --- | --- | --- | --- | --- |
|  | **FP-CIT Metric** | **AUC (95% CI)** | **Sensitivity** | **Specificity** | **PPV** | **LR+** | **LR−** | **TP** | **FN** | **FP** | **TN** | **p** |
| **CBD (n=43)  vs. PD (n=487)** | SBR Ventral Striatum (I) | 0.511 (0.421–0.602) | 0.651 | 0.431 | 0.092 | 1.14 | 0.81 | 28 | 15 | 277 | 210 | 0.805 |
|  | SBR Ventral Striatum (C) | 0.492 (0.409–0.576) | 0.860 | 0.203 | 0.087 | 1.08 | 0.69 | 37 | 6 | 388 | 99 | 0.87 |
|  | SBR Putamen (ant.) (I) | 0.619 (0.538–0.701) | 0.767 | 0.460 | 0.111 | 1.42 | 0.51 | 33 | 10 | 263 | 224 | 0.009 |
|  | SBR Putamen (ant.) (C) | 0.629 (0.549–0.709) | 0.791 | 0.470 | 0.116 | 1.49 | 0.45 | 34 | 9 | 258 | 229 | 0.005 |
|  | SBR Putamen (post.) (I) | 0.722 (0.637–0.807) | 0.744 | 0.630 | 0.151 | 2.01 | 0.41 | 32 | 11 | 180 | 307 | <0.001 |
|  | SBR Putamen (post.) (C) | 0.738 (0.661–0.815) | 0.860 | 0.540 | 0.142 | 1.87 | 0.26 | 37 | 6 | 224 | 263 | <0.001 |
|  | SBR Caudate (I) | 0.526 (0.435–0.617) | 0.465 | 0.637 | 0.102 | 1.28 | 0.84 | 20 | 23 | 177 | 310 | 0.569 |
|  | SBR Caudate (C) | 0.494 (0.411–0.578) | 0.512 | 0.556 | 0.092 | 1.15 | 0.88 | 22 | 21 | 216 | 271 | 0.901 |
|  | Cau-Put (ant.) ratio | 0.842 (0.788–0.896) | 0.977 | 0.606 | 0.179 | 2.48 | 0.04 | 42 | 1 | 192 | 295 | <0.001 |
|  | Cau-Put (post.) ratio | 0.871 (0.811–0.930) | 0.791 | 0.875 | 0.358 | 6.31 | 0.24 | 34 | 9 | 61 | 426 | <0.001 |
|  | AI Ventral Striatum | 0.541 (0.443–0.639) | 0.558 | 0.583 | 0.106 | 1.34 | 0.76 | 24 | 19 | 203 | 284 | 0.371 |
|  | AI Putamen (ant.) | 0.564 (0.481–0.647) | 0.953 | 0.191 | 0.094 | 1.18 | 0.24 | 41 | 2 | 394 | 93 | 0.164 |
|  | AI Putamen (post.) | 0.497 (0.407–0.587) | 0.884 | 0.187 | 0.088 | 1.09 | 0.62 | 38 | 5 | 396 | 91 | 0.942 |
|  | AI Caudate | 0.553 (0.454–0.651) | 0.256 | 0.871 | 0.149 | 1.98 | 0.85 | 11 | 32 | 63 | 424 | 0.251 |
|  | SBR Cognitive (I) | 0.562 (0.475–0.650) | 0.651 | 0.505 | 0.104 | 1.32 | 0.69 | 28 | 15 | 241 | 246 | 0.176 |
|  | SBR Cognitive (C) | 0.578 (0.497–0.660) | 0.814 | 0.413 | 0.109 | 1.39 | 0.45 | 35 | 8 | 286 | 201 | 0.089 |
|  | SBR Executive (I) | 0.562 (0.474–0.650) | 0.767 | 0.400 | 0.102 | 1.28 | 0.58 | 33 | 10 | 292 | 195 | 0.176 |
|  | SBR Executive (C) | 0.581 (0.499–0.663) | 0.791 | 0.419 | 0.107 | 1.36 | 0.5 | 34 | 9 | 283 | 204 | 0.08 |
|  | SBR Limbic (I) | 0.563 (0.473–0.652) | 0.651 | 0.487 | 0.101 | 1.27 | 0.72 | 28 | 15 | 250 | 237 | 0.173 |
|  | SBR Limbic (C) | 0.568 (0.487–0.650) | 0.814 | 0.388 | 0.105 | 1.33 | 0.48 | 35 | 8 | 298 | 189 | 0.137 |
|  | SBR Sensorimotor (I) | 0.684 (0.598–0.770) | 0.698 | 0.610 | 0.136 | 1.79 | 0.5 | 30 | 13 | 190 | 297 | <0.001 |
|  | SBR Sensorimotor (C) | 0.707 (0.628–0.785) | 0.791 | 0.573 | 0.140 | 1.85 | 0.37 | 34 | 9 | 208 | 279 | <0.001 |
|  | Sen-Cog ratio | 0.821 (0.748–0.895) | 0.605 | 0.926 | 0.419 | 8.18 | 0.43 | 26 | 17 | 36 | 451 | <0.001 |
|  | Sen-Exe ratio | 0.820 (0.753–0.887) | 0.651 | 0.877 | 0.318 | 5.29 | 0.4 | 28 | 15 | 60 | 427 | <0.001 |
|  | Sen-Lim ratio | 0.810 (0.727–0.893) | 0.814 | 0.723 | 0.206 | 2.94 | 0.26 | 35 | 8 | 135 | 352 | <0.001 |
|  | AI Cognitive | 0.506 (0.410–0.601) | 0.465 | 0.628 | 0.100 | 1.25 | 0.85 | 20 | 23 | 181 | 306 | 0.9 |
|  | AI Executive | 0.502 (0.410–0.594) | 0.302 | 0.754 | 0.098 | 1.23 | 0.93 | 13 | 30 | 120 | 367 | 0.962 |
|  | AI Limbic | 0.553 (0.451–0.655) | 0.395 | 0.754 | 0.124 | 1.6 | 0.8 | 17 | 26 | 120 | 367 | 0.252 |
|  | AI Sensorimotor | 0.508 (0.419–0.598) | 0.116 | 0.953 | 0.179 | 2.46 | 0.93 | 5 | 38 | 23 | 464 | 0.858 |
| **MSA-P (n=37) vs. PD (n=487)** | SBR Ventral Striatum (I) | 0.727 (0.654–0.800) | 0.973 | 0.384 | 0.107 | 1.58 | 0.07 | 36 | 1 | 300 | 187 | <0.001 |
|  | SBR Ventral Striatum (C) | 0.715 (0.640–0.790) | 0.838 | 0.501 | 0.113 | 1.68 | 0.32 | 31 | 6 | 243 | 244 | <0.001 |
|  | SBR Putamen (ant.) (I) | 0.687 (0.612–0.763) | 0.811 | 0.573 | 0.126 | 1.9 | 0.33 | 30 | 7 | 208 | 279 | <0.001 |
|  | SBR Putamen (ant.) (C) | 0.692 (0.619–0.766) | 0.730 | 0.618 | 0.127 | 1.91 | 0.44 | 27 | 10 | 186 | 301 | <0.001 |
|  | SBR Putamen (post.) (I) | 0.669 (0.576–0.762) | 0.703 | 0.626 | 0.125 | 1.88 | 0.47 | 26 | 11 | 182 | 305 | <0.001 |
|  | SBR Putamen (post.) (C) | 0.642 (0.554–0.729) | 0.784 | 0.472 | 0.101 | 1.49 | 0.46 | 29 | 8 | 257 | 230 | 0.004 |
|  | SBR Caudate (I) | 0.699 (0.618–0.781) | 0.703 | 0.616 | 0.122 | 1.83 | 0.48 | 26 | 11 | 187 | 300 | <0.001 |
|  | SBR Caudate (C) | 0.678 (0.598–0.758) | 0.730 | 0.581 | 0.117 | 1.74 | 0.47 | 27 | 10 | 204 | 283 | <0.001 |
|  | Cau-Put (ant.) ratio | 0.496 (0.392–0.600) | 0.324 | 0.772 | 0.098 | 1.42 | 0.88 | 12 | 25 | 111 | 376 | 0.935 |
|  | Cau-Put (post.) ratio | 0.579 (0.473–0.685) | 0.459 | 0.731 | 0.115 | 1.71 | 0.74 | 17 | 20 | 131 | 356 | 0.11 |
|  | AI Ventral Striatum | 0.515 (0.419–0.612) | 0.838 | 0.257 | 0.079 | 1.13 | 0.63 | 31 | 6 | 362 | 125 | 0.755 |
|  | AI Putamen (ant.) | 0.508 (0.401–0.614) | 0.243 | 0.881 | 0.134 | 2.04 | 0.86 | 9 | 28 | 58 | 429 | 0.879 |
|  | AI Putamen (post.) | 0.537 (0.412–0.663) | 0.378 | 0.883 | 0.197 | 3.23 | 0.7 | 14 | 23 | 57 | 430 | 0.451 |
|  | AI Caudate | 0.573 (0.467–0.679) | 0.270 | 0.916 | 0.196 | 3.21 | 0.8 | 10 | 27 | 41 | 446 | 0.137 |
|  | SBR Cognitive (I) | 0.706 (0.630–0.782) | 0.649 | 0.725 | 0.152 | 2.36 | 0.48 | 24 | 13 | 134 | 353 | <0.001 |
|  | SBR Cognitive (C) | 0.695 (0.619–0.771) | 0.649 | 0.700 | 0.141 | 2.16 | 0.5 | 24 | 13 | 146 | 341 | <0.001 |
|  | SBR Executive (I) | 0.698 (0.620–0.776) | 0.622 | 0.715 | 0.142 | 2.18 | 0.53 | 23 | 14 | 139 | 348 | <0.001 |
|  | SBR Executive (C) | 0.688 (0.612–0.764) | 0.730 | 0.598 | 0.121 | 1.81 | 0.45 | 27 | 10 | 196 | 291 | <0.001 |
|  | SBR Limbic (I) | 0.718 (0.644–0.792) | 0.649 | 0.698 | 0.140 | 2.15 | 0.5 | 24 | 13 | 147 | 340 | <0.001 |
|  | SBR Limbic (C) | 0.708 (0.634–0.783) | 0.622 | 0.723 | 0.146 | 2.24 | 0.52 | 23 | 14 | 135 | 352 | <0.001 |
|  | SBR Sensorimotor (I) | 0.671 (0.583–0.759) | 0.703 | 0.632 | 0.127 | 1.91 | 0.47 | 26 | 11 | 179 | 308 | <0.001 |
|  | SBR Sensorimotor (C) | 0.647 (0.563–0.732) | 0.757 | 0.532 | 0.109 | 1.62 | 0.46 | 28 | 9 | 228 | 259 | 0.003 |
|  | Sen-Cog ratio | 0.580 (0.484–0.676) | 0.270 | 0.871 | 0.137 | 2.09 | 0.84 | 10 | 27 | 63 | 424 | 0.105 |
|  | Sen-Exe ratio | 0.591 (0.497–0.685) | 0.378 | 0.782 | 0.117 | 1.74 | 0.79 | 14 | 23 | 106 | 381 | 0.065 |
|  | Sen-Lim ratio | 0.557 (0.460–0.654) | 0.676 | 0.468 | 0.088 | 1.27 | 0.69 | 25 | 12 | 259 | 228 | 0.244 |
|  | AI Cognitive | 0.534 (0.420–0.648) | 0.297 | 0.877 | 0.155 | 2.41 | 0.8 | 11 | 26 | 60 | 427 | 0.492 |
|  | AI Executive | 0.555 (0.448–0.662) | 0.270 | 0.916 | 0.196 | 3.21 | 0.8 | 10 | 27 | 41 | 446 | 0.265 |
|  | AI Limbic | 0.466 (0.369–0.562) | 0.649 | 0.444 | 0.081 | 1.17 | 0.79 | 24 | 13 | 271 | 216 | 0.486 |
|  | AI Sensorimotor | 0.601 (0.491–0.711) | 0.351 | 0.922 | 0.255 | 4.5 | 0.7 | 13 | 24 | 38 | 449 | 0.04 |
|  |  |  |  |  |  |  |  |  |  |  |  |  |
| **MSA-C (n=12) vs. PD (n=487)*** | SBR Ventral Striatum (I) | 0.702 (0.560–0.845) | 0.917 | 0.513 | 0.044 | 1.88 | 0.16 | 11 | 1 | 237 | 250 | 0.017 |
|  | SBR Ventral Striatum (C) | 0.607 (0.470–0.744) | 0.917 | 0.474 | 0.041 | 1.74 | 0.18 | 11 | 1 | 256 | 231 | 0.205 |
|  | SBR Putamen (ant.) (I) | 0.795 (0.683–0.907) | 0.917 | 0.692 | 0.068 | 2.98 | 0.12 | 11 | 1 | 150 | 337 | <0.001 |
|  | SBR Putamen (ant.) (C) | 0.699 (0.590–0.809) | 0.917 | 0.524 | 0.045 | 1.92 | 0.16 | 11 | 1 | 232 | 255 | 0.018 |
|  | SBR Putamen (post.) (I) | 0.921 (0.845–0.998) | 0.917 | 0.862 | 0.141 | 6.66 | 0.1 | 11 | 1 | 67 | 420 | <0.001 |
|  | SBR Putamen (post.) (C) | 0.866 (0.764–0.968) | 0.833 | 0.836 | 0.111 | 5.07 | 0.2 | 10 | 2 | 80 | 407 | <0.001 |
|  | SBR Caudate (I) | 0.758 (0.615–0.901) | 0.917 | 0.655 | 0.061 | 2.66 | 0.13 | 11 | 1 | 168 | 319 | 0.002 |
|  | SBR Caudate (C) | 0.687 (0.538–0.836) | 0.833 | 0.595 | 0.048 | 2.06 | 0.28 | 10 | 2 | 197 | 290 | 0.027 |
|  | Cau-Put (ant.) ratio | 0.620 (0.505–0.734) | 1.000 | 0.376 | 0.038 | 1.6 | 0 | 12 | 0 | 304 | 183 | 0.156 |
|  | Cau-Put (post.) ratio | 0.914 (0.881–0.946) | 1.000 | 0.848 | 0.140 | 6.58 | 0 | 12 | 0 | 74 | 413 | <0.001 |
|  | AI Ventral Striatum | 0.615 (0.484–0.746) | 0.667 | 0.581 | 0.038 | 1.59 | 0.57 | 8 | 4 | 204 | 283 | 0.173 |
|  | AI Putamen (ant.) | 0.671 (0.530–0.811) | 0.833 | 0.480 | 0.038 | 1.6 | 0.35 | 10 | 2 | 253 | 234 | 0.043 |
|  | AI Putamen (post.) | 0.822 (0.751–0.893) | 0.917 | 0.667 | 0.064 | 2.76 | 0.12 | 11 | 1 | 162 | 325 | <0.001 |
|  | AI Caudate | 0.766 (0.659–0.873) | 0.917 | 0.630 | 0.058 | 2.48 | 0.13 | 11 | 1 | 180 | 307 | 0.002 |
|  | SBR Cognitive (I) | 0.784 (0.653–0.915) | 0.833 | 0.768 | 0.081 | 3.59 | 0.22 | 10 | 2 | 113 | 374 | <0.001 |
|  | SBR Cognitive (C) | 0.686 (0.555–0.818) | 0.917 | 0.542 | 0.047 | 2 | 0.15 | 11 | 1 | 223 | 264 | 0.027 |
|  | SBR Executive (I) | 0.794 (0.663–0.926) | 0.833 | 0.766 | 0.081 | 3.56 | 0.22 | 10 | 2 | 114 | 373 | <0.001 |
|  | SBR Executive (C) | 0.705 (0.570–0.840) | 0.917 | 0.532 | 0.046 | 1.96 | 0.16 | 11 | 1 | 228 | 259 | 0.015 |
|  | SBR Limbic (I) | 0.748 (0.621–0.874) | 0.917 | 0.602 | 0.054 | 2.3 | 0.14 | 11 | 1 | 194 | 293 | 0.003 |
|  | SBR Limbic (C) | 0.636 (0.507–0.764) | 0.917 | 0.540 | 0.047 | 1.99 | 0.15 | 11 | 1 | 224 | 263 | 0.108 |
|  | SBR Sensorimotor (I) | 0.894 (0.795–0.993) | 0.917 | 0.830 | 0.117 | 5.38 | 0.1 | 11 | 1 | 83 | 404 | <0.001 |
|  | SBR Sensorimotor (C) | 0.846 (0.738–0.954) | 0.917 | 0.741 | 0.080 | 3.54 | 0.11 | 11 | 1 | 126 | 361 | <0.001 |
|  | Sen-Cog ratio | 0.935 (0.902–0.967) | 0.917 | 0.889 | 0.169 | 8.27 | 0.09 | 11 | 1 | 54 | 433 | <0.001 |
|  | Sen-Exe ratio | 0.897 (0.851–0.943) | 1.000 | 0.717 | 0.080 | 3.53 | 0 | 12 | 0 | 138 | 349 | <0.001 |
|  | Sen-Lim ratio | 0.955 (0.931–0.978) | 1.000 | 0.875 | 0.164 | 7.98 | 0 | 12 | 0 | 61 | 426 | <0.001 |
|  | AI Cognitive | 0.797 (0.715–0.878) | 1.000 | 0.602 | 0.058 | 2.51 | 0 | 12 | 0 | 194 | 293 | <0.001 |
|  | AI Executive | 0.748 (0.660–0.836) | 0.917 | 0.591 | 0.052 | 2.24 | 0.14 | 11 | 1 | 199 | 288 | 0.003 |
|  | AI Limbic | 0.766 (0.683–0.850) | 1.000 | 0.573 | 0.055 | 2.34 | 0 | 12 | 0 | 208 | 279 | 0.002 |
|  | AI Sensorimotor | 0.776 (0.699–0.852) | 1.000 | 0.536 | 0.050 | 2.15 | 0 | 12 | 0 | 226 | 261 | 0.001 |
|  |  |  |  |  |  |  |  |  |  |  |  |  |
| **PSP (n=127) vs. PD (n=487)** | SBR Ventral Striatum (I) | 0.593 (0.538–0.648) | 0.606 | 0.550 | 0.260 | 1.35 | 0.72 | 77 | 50 | 219 | 268 | 0.001 |
|  | SBR Ventral Striatum (C) | 0.606 (0.552–0.659) | 0.630 | 0.540 | 0.263 | 1.37 | 0.69 | 80 | 47 | 224 | 263 | <0.001 |
|  | SBR Putamen (ant.) (I) | 0.540 (0.485–0.594) | 0.677 | 0.425 | 0.235 | 1.18 | 0.76 | 86 | 41 | 280 | 207 | 0.169 |
|  | SBR Putamen (ant.) (C) | 0.565 (0.511–0.619) | 0.701 | 0.423 | 0.241 | 1.21 | 0.71 | 89 | 38 | 281 | 206 | 0.024 |
|  | SBR Putamen (post.) (I) | 0.539 (0.480–0.598) | 0.409 | 0.700 | 0.263 | 1.37 | 0.84 | 52 | 75 | 146 | 341 | 0.178 |
|  | SBR Putamen (post.) (C) | 0.501 (0.443–0.559) | 0.575 | 0.476 | 0.223 | 1.1 | 0.89 | 73 | 54 | 255 | 232 | 0.976 |
|  | SBR Caudate (I) | 0.639 (0.586–0.692) | 0.803 | 0.427 | 0.268 | 1.4 | 0.46 | 102 | 25 | 279 | 208 | <0.001 |
|  | SBR Caudate (C) | 0.653 (0.602–0.704) | 0.756 | 0.487 | 0.277 | 1.47 | 0.5 | 96 | 31 | 250 | 237 | <0.001 |
|  | Cau-Put (ant.) ratio | 0.783 (0.737–0.829) | 0.819 | 0.641 | 0.373 | 2.28 | 0.28 | 104 | 23 | 175 | 312 | <0.001 |
|  | Cau-Put (post.) ratio | 0.809 (0.764–0.853) | 0.780 | 0.731 | 0.430 | 2.9 | 0.3 | 99 | 28 | 131 | 356 | <0.001 |
|  | AI Ventral Striatum | 0.479 (0.424–0.535) | 0.583 | 0.474 | 0.224 | 1.11 | 0.88 | 74 | 53 | 256 | 231 | 0.476 |
|  | AI Putamen (ant.) | 0.548 (0.492–0.604) | 0.394 | 0.702 | 0.256 | 1.32 | 0.86 | 50 | 77 | 145 | 342 | 0.096 |
|  | AI Putamen (post.) | 0.611 (0.562–0.660) | 0.732 | 0.485 | 0.270 | 1.42 | 0.55 | 93 | 34 | 251 | 236 | <0.001 |
|  | AI Caudate | 0.514 (0.456–0.573) | 0.425 | 0.651 | 0.241 | 1.22 | 0.88 | 54 | 73 | 170 | 317 | 0.618 |
|  | SBR Cognitive (I) | 0.580 (0.526–0.634) | 0.795 | 0.351 | 0.242 | 1.23 | 0.58 | 101 | 26 | 316 | 171 | 0.005 |
|  | SBR Cognitive (C) | 0.602 (0.549–0.655) | 0.772 | 0.407 | 0.253 | 1.3 | 0.56 | 98 | 29 | 289 | 198 | <0.001 |
|  | SBR Executive (I) | 0.584 (0.530–0.638) | 0.803 | 0.357 | 0.246 | 1.25 | 0.55 | 102 | 25 | 313 | 174 | 0.004 |
|  | SBR Executive (C) | 0.607 (0.554–0.660) | 0.787 | 0.405 | 0.256 | 1.32 | 0.53 | 100 | 27 | 290 | 197 | <0.001 |
|  | SBR Limbic (I) | 0.568 (0.513–0.623) | 0.740 | 0.398 | 0.243 | 1.23 | 0.65 | 94 | 33 | 293 | 194 | 0.019 |
|  | SBR Limbic (C) | 0.586 (0.532–0.640) | 0.756 | 0.400 | 0.247 | 1.26 | 0.61 | 96 | 31 | 292 | 195 | 0.003 |
|  | SBR Sensorimotor (I) | 0.495 (0.437–0.553) | 0.370 | 0.684 | 0.234 | 1.17 | 0.92 | 47 | 80 | 154 | 333 | 0.861 |
|  | SBR Sensorimotor (C) | 0.473 (0.416–0.530) | 0.457 | 0.565 | 0.215 | 1.05 | 0.96 | 58 | 69 | 212 | 275 | 0.343 |
|  | Sen-Cog ratio | 0.716 (0.665–0.768) | 0.472 | 0.848 | 0.448 | 3.11 | 0.62 | 60 | 67 | 74 | 413 | <0.001 |
|  | Sen-Exe ratio | 0.754 (0.707–0.802) | 0.756 | 0.641 | 0.354 | 2.1 | 0.38 | 96 | 31 | 175 | 312 | <0.001 |
|  | Sen-Lim ratio | 0.632 (0.573–0.691) | 0.417 | 0.844 | 0.411 | 2.67 | 0.69 | 53 | 74 | 76 | 411 | <0.001 |
|  | AI Cognitive | 0.543 (0.489–0.597) | 0.780 | 0.337 | 0.235 | 1.18 | 0.65 | 99 | 28 | 323 | 164 | 0.135 |
|  | AI Executive | 0.547 (0.493–0.601) | 0.701 | 0.437 | 0.245 | 1.25 | 0.68 | 89 | 38 | 274 | 213 | 0.104 |
|  | AI Limbic | 0.534 (0.476–0.592) | 0.591 | 0.499 | 0.235 | 1.18 | 0.82 | 75 | 52 | 244 | 243 | 0.237 |
|  | AI Sensorimotor | 0.545 (0.493–0.596) | 0.780 | 0.368 | 0.243 | 1.23 | 0.6 | 99 | 28 | 308 | 179 | 0.121 |
| Each atypical parkinsonian syndrome (APS) group was treated as the positive class in all comparisons. The ROC direction was determined automatically to ensure AUC ≥ 0.5. Optimal cut-offs were selected using the Youden index (J = sensitivity + specificity − 1). AUC 95% confidence intervals and p-values were computed using the DeLong method. Sensitivity and specificity were verified directly from classification at the reported cut-off. Positive predictive value (PPV) is reported positive and negative likelihood ratios (LR+ and LR−) as prevalence-independent measures of diagnostic test performance: LR+ = sensitivity / (1 − specificity); LR− = (1 − sensitivity) / specificity. *For MSA-C vs. PD comparisons (n = 12), sensitivity = 1.000 and LR− = 0.00 indicate that all 12 MSA-C cases fell above the optimal threshold and should NOT be interpreted as evidence of perfect sensitivity or perfect rule-out performance. The exact binomial 95% CI for sensitivity = 1.000 with n = 12 extends down to 0.74 (Clopper-Pearson), indicating that true sensitivity may be substantially lower in a larger sample. The TP/FN counts confirm this: FN = 0 in these cases reflects only that no MSA-C case fell below the threshold in this specific sample.  Abbreviations: AUC = area under the curve; CBD = corticobasal degeneration; CI = confidence interval; LR+ = positive likelihood ratio; FN = false negative; FP = false positive; LR− = negative likelihood ratio; MSA-P = multiple system atrophy parkinsonian type; MSA-C = multiple system atrophy cerebellar type; PD = Parkinson's disease; PPV = positive predictive value; PSP = progressive supranuclear palsy; TN = true negative; TP = true positive. | | | | | | | | | | | | |

*The APS group was treated as the positive class in all comparisons. ROC direction is determined automatically to ensure AUC ≥ 0.5. Cut-offs selected using the Youden index. AUC 95% CIs by DeLong method. p-values by Wilcoxon rank-sum test. TP = true positives; FN = false negatives; FP = false positives; TN = true negatives at the Youden-optimal threshold. For MSA-C vs. PD comparisons (n = 12), sensitivity = 1.000 and LR− = 0.00 indicate that all 12 MSA-C cases fell above the optimal threshold and should NOT be interpreted as evidence of perfect sensitivity or perfect rule-out performance. The exact binomial 95% CI for sensitivity = 1.000 with n = 12 extends down to 0.74 (Clopper-Pearson), indicating that true sensitivity may be substantially lower in a larger sample. The TP/FN counts confirm this: FN = 0 in these cases reflects only that no MSA-C case fell below the threshold in this specific sample. Abbreviations: AUC = area under the curve; CBD = corticobasal degeneration; CI = confidence interval; LR = likelihood ratio; MSA-P = multiple system atrophy parkinsonian type; MSA-C = multiple system atrophy cerebellar type; PD = Parkinson’s disease; PPV = positive predictive value; PSP = progressive supranuclear palsy.*

| **Table S2. Pair-wise comparisons of regional FP-CIT SPECT metrics among atypical parkinsonian syndromes** | | | | | | | | | | | | | | | |
| --- | --- | --- | --- | --- | --- | --- | --- | --- | --- | --- | --- | --- | --- | --- | --- |
|  | ***CBD vs. MSA-P*** | | | ***CBD vs. MSA-C*** | | | ***CBD vs. PSP*** | | | ***MSA-P vs. PSP*** | | | ***MSA-C vs. PSP*** | | |
|  | β (CI) | p | d | β (CI) | p | d | β (CI) | p | d | β (CI) | p | d | β (CI) | p | d |
| ***Anatomical striatal ROIs*** | | | | | | | | | | | | | | | |
| SBR Ventral Striatum (I) | 0.4 (0.2–0.6) | **<0.001** | **0.9** | -0.3 (-0.7–0) | 0.051 | 0.7 | 0.2 (0–0.3) | 0.051 | 0.3 | -0.2 (-0.4–-0.1) | **0.007** | **0.5** | 0.5 (0.2–0.8) | **0.004** | **1** |
| SBR Ventral Striatum (C) | 0.4 (0.2–0.6) | **<0.001** | **0.9** | -0.2 (-0.5–0.1) | 0.294 | 0.4 | 0.2 (0.1–0.4) | **0.021** | **0.4** | -0.2 (-0.4–0) | **0.022** | **0.4** | 0.4 (0.1–0.7) | **0.021** | **0.7** |
| SBR Putamen (ant.) (I) | 0.7 (0.5–1) | **<0.001** | **1.3** | -0.4 (-0.8–-0.1) | **0.02** | **0.7** | 0.4 (0.2–0.6) | **<0.001** | **0.6** | -0.4 (-0.6–-0.2) | **<0.001** | **0.6** | 0.8 (0.5–1.2) | **<0.001** | **1.3** |
| SBR Putamen (ant.) (C) | 0.8 (0.5–1) | **<0.001** | **1.3** | -0.2 (-0.5–0.2) | 0.302 | 0.3 | 0.5 (0.3–0.7) | **<0.001** | **0.7** | -0.3 (-0.5–-0.1) | **0.006** | **0.5** | 0.7 (0.3–1) | **<0.001** | **1** |
| SBR Putamen (post.) (I) | 0.6 (0.4–0.8) | **<0.001** | **1.3** | -0.4 (-0.7–-0.1) | **0.003** | **0.8** | 0.3 (0.1–0.5) | **<0.001** | **0.7** | -0.3 (-0.4–-0.1) | **<0.001** | **0.7** | 0.7 (0.5–1) | **<0.001** | **1.6** |
| SBR Putamen (post.) (C) | 0.7 (0.5–0.9) | **<0.001** | **1.4** | -0.3 (-0.6–0) | 0.086 | 0.5 | 0.4 (0.2–0.6) | **<0.001** | **0.8** | -0.2 (-0.4–-0.1) | **0.004** | **0.5** | 0.7 (0.4–1) | **<0.001** | **1.4** |
| SBR Caudate (I) | 0.3 (0.1–0.5) | **0.007** | **0.7** | -0.5 (-0.8–-0.1) | **0.008** | **1** | 0.2 (0–0.4) | **0.017** | **0.4** | -0.1 (-0.3–0.1) | 0.224 | 0.2 | 0.7 (0.4–1) | **<0.001** | **1.5** |
| SBR Caudate (C) | 0.3 (0.1–0.5) | **0.002** | **0.7** | -0.3 (-0.6–0) | 0.09 | 0.7 | 0.3 (0.1–0.4) | **<0.001** | **0.6** | 0 (-0.2–0.1) | 0.591 | 0.1 | 0.6 (0.3–0.9) | **<0.001** | **1.2** |
| Cau-Put (ant.) ratio | 0.8 (0.8–0.9) | **<0.001** | **0.9** | 0.9 (0.8–1) | 0.184 | 0.5 | 1 (0.9–1) | 0.481 | 0.1 | 1.2 (1.1–1.3) | **<0.001** | **0.8** | 1.1 (1–1.2) | 0.235 | 0.4 |
| Cau-Put (post.) ratio | 0.8 (0.7–0.9) | **0.016** | **0.6** | 1.1 (0.9–1.3) | 0.715 | 0.2 | 1 (0.9–1.1) | 0.992 | 0 | 1.2 (1.1–1.4) | **0.006** | **0.6** | 0.9 (0.8–1.1) | 0.715 | 0.2 |
| AI Ventral Striatum | 1.1 (0.8–1.7) | 0.625 | 0.1 | 1.6 (0.9–2.9) | 0.3 | 0.5 | 0.9 (0.7–1.3) | 0.625 | 0.1 | 0.8 (0.6–1.1) | 0.377 | 0.2 | 0.6 (0.3–1) | 0.249 | 0.6 |
| AI Putamen (ant.) | 0.7 (0.5–1) | 0.166 | 0.5 | 1.4 (0.8–2.5) | 0.295 | 0.4 | 0.9 (0.6–1.2) | 0.382 | 0.2 | 1.3 (0.9–1.8) | 0.166 | 0.3 | 0.6 (0.4–1) | 0.166 | 0.5 |
| AI Putamen (post.) | 0.8 (0.6–1.1) | 0.104 | 0.4 | 3.2 (2.1–5) | **<0.001** | **1.7** | 1.4 (1.1–1.8) | **0.01** | **0.5** | 1.8 (1.4–2.3) | **<0.001** | **0.8** | 0.4 (0.3–0.7) | **<0.001** | **1.2** |
| AI Caudate | 0.8 (0.5–1.3) | 0.399 | 0.2 | 3.1 (1.6–5.9) | **0.003** | **1.1** | 1.2 (0.8–1.6) | 0.399 | 0.1 | 1.4 (1–2.1) | 0.101 | 0.4 | 0.4 (0.2–0.7) | **0.003** | **1** |
| ***Functional striatal ROIs*** | | | | | | | | | | | | | | | |
| SBR Cognitive (I) | 0.5 (0.3–0.7) | **<0.001** | **1.1** | -0.4 (-0.8–-0.1) | **0.012** | **0.8** | 0.3 (0.1–0.5) | **0.005** | **0.5** | -0.2 (-0.4–-0.1) | **0.008** | **0.5** | 0.7 (0.4–1) | **<0.001** | **1.4** |
| SBR Cognitive (C) | 0.6 (0.3–0.8) | **<0.001** | **1.1** | -0.2 (-0.6–0.1) | 0.174 | 0.4 | 0.4 (0.2–0.5) | **<0.001** | **0.7** | -0.2 (-0.4–0) | 0.051 | 0.4 | 0.6 (0.3–0.9) | **<0.001** | **1.1** |
| SBR Executive (I) | 0.5 (0.3–0.7) | **<0.001** | **1** | -0.5 (-0.9–-0.1) | **0.011** | **0.9** | 0.3 (0.1–0.5) | **0.004** | **0.6** | -0.2 (-0.4–0) | **0.012** | **0.4** | 0.8 (0.4–1.1) | **<0.001** | **1.5** |
| SBR Executive (C) | 0.6 (0.3–0.8) | **<0.001** | **1.1** | -0.3 (-0.6–0.1) | 0.126 | 0.5 | 0.4 (0.2–0.6) | **<0.001** | **0.7** | -0.2 (-0.4–0) | 0.088 | 0.3 | 0.7 (0.3–1) | **<0.001** | **1.2** |
| SBR Limbic (I) | 0.5 (0.3–0.7) | **<0.001** | **1.1** | -0.3 (-0.6–0) | **0.032** | **0.7** | 0.2 (0.1–0.4) | **0.012** | **0.5** | -0.3 (-0.4–-0.1) | **0.002** | **0.6** | 0.6 (0.3–0.9) | **<0.001** | **1.1** |
| SBR Limbic (C) | 0.5 (0.3–0.7) | **<0.001** | **1.1** | -0.1 (-0.4–0.2) | 0.377 | 0.3 | 0.3 (0.1–0.5) | **0.002** | **0.6** | -0.2 (-0.4–-0.1) | **0.013** | **0.5** | 0.4 (0.1–0.7) | **0.005** | **0.8** |
| SBR Sensorimotor (I) | 0.5 (0.4–0.7) | **<0.001** | **1.3** | -0.4 (-0.7–-0.1) | **0.004** | **0.9** | 0.3 (0.1–0.5) | **<0.001** | **0.7** | -0.2 (-0.4–-0.1) | **<0.001** | **0.6** | 0.7 (0.5–0.9) | **<0.001** | **1.6** |
| SBR Sensorimotor (C) | 0.6 (0.4–0.8) | **<0.001** | **1.3** | -0.3 (-0.5–0) | 0.055 | 0.6 | 0.4 (0.2–0.6) | **<0.001** | **0.8** | -0.2 (-0.4–0) | **0.014** | **0.4** | 0.7 (0.4–0.9) | **<0.001** | **1.4** |
| Sen-Cog ratio | 1.1 (1–1.2) | **0.015** | **0.7** | 1 (0.9–1.1) | 0.466 | 0.2 | 1.1 (1–1.1) | 0.085 | 0.3 | 0.9 (0.9–1) | 0.085 | 0.3 | 1.1 (1–1.2) | 0.085 | 0.6 |
| Sen-Exe ratio | 1.1 (1–1.2) | 0.072 | 0.6 | 1 (0.9–1.1) | 0.694 | 0.1 | 1 (1–1.1) | 0.376 | 0.2 | 0.9 (0.9–1) | 0.122 | 0.4 | 1.1 (1–1.2) | 0.376 | 0.3 |
| Sen-Lim ratio | 1.2 (1–1.3) | **0.026** | **0.6** | 0.9 (0.8–1.1) | 0.297 | 0.3 | 1.1 (1–1.2) | **0.049** | **0.4** | 0.9 (0.9–1) | 0.234 | 0.2 | 1.2 (1–1.4) | **0.041** | **0.7** |
| AI Cognitive | 0.8 (0.5–1.1) | 0.183 | 0.3 | 3.2 (1.9–5.4) | **<0.001** | **1.4** | 1.1 (0.9–1.5) | 0.341 | 0.2 | 1.5 (1.1–2) | **0.014** | **0.5** | 0.4 (0.2–0.6) | **<0.001** | **1.2** |
| AI Executive | 0.7 (0.5–1) | 0.089 | 0.4 | 2.5 (1.5–4.4) | **0.004** | **1.1** | 1.1 (0.8–1.5) | 0.49 | 0.1 | 1.6 (1.1–2.1) | **0.008** | **0.5** | 0.4 (0.3–0.7) | **0.004** | **1** |
| AI Limbic | 0.8 (0.5–1.3) | 0.472 | 0.2 | 2.6 (1.4–4.8) | **0.005** | **1** | 1 (0.7–1.4) | 0.919 | 0 | 1.2 (0.9–1.7) | 0.412 | 0.2 | 0.4 (0.2–0.7) | **0.005** | **1** |
| AI Sensorimotor | 0.8 (0.6–1) | 0.088 | 0.4 | 2.8 (1.8–4.3) | **<0.001** | **1.5** | 1.3 (1–1.6) | 0.074 | 0.3 | 1.6 (1.3–2.1) | **<0.001** | **0.7** | 0.4 (0.3–0.7) | **<0.001** | **1.2** |
| Values represent standardized regression coefficients (β) with 95% confidence intervals (CI), *p*-values, and Cohen’s *d* effect sizes for pairwise group comparisons of FP-CIT specific binding ratios (SBRs), ratios, and asymmetry indices (AI) in anatomical and functional striatal regions of interest (ROIs). Comparisons were adjusted for age, sex, and disease duration. For SBR metrics, β denotes the absolute mean difference between each APS group and the PD group, adjusted for age, sex, and disease duration. For ratio and asymmetry index (AI) variables, generalized linear models with a Gamma distribution and log-link function were fitted. In these cases, the reported β indicates the ratio of means, defined as the mean value in the APS group divided by the mean value in the PD group. Values less than 1 indicate lower values in the APS group compared to PD, values greater than 1 indicate higher values, and values near 1 suggest no meaningful difference. Confidence intervals are reported on the same scale. Abbreviations: AI = asymmetry index; (ant.) = anterior; (C) = contralateral hemisphere relative to the most affected side; CBD = corticobasal degeneration; (I) = ipsilateral hemisphere relative to the most affected side; MSA-P = multiple system atrophy, parkinsonian type; MSA-C = multiple system atrophy, cerebellar type; PSP = progressive supranuclear palsy; SBR = standard binding ratio; (post) = posterior. | | | | | | | | | | | | | | | |

| **Table S3. Diagnostic performance of FP-CIT metrics for distinguishing atypical parkinsonian syndromes from each other** | | | | | | | | | | | | |
| --- | --- | --- | --- | --- | --- | --- | --- | --- | --- | --- | --- | --- |
|  | **FP-CIT Metrics** | **AUC (95% CI)** | **Sensitivity** | **Specificity** | **PPV** | **LR+** | **LR−** | **TP** | **FN** | **FP** | **TN** | **p** |
| **CBD (n=43)**  **vs. MSA-P (n=37)** | SBR Ventral Striatum (I) | 0.736 (0.627–0.845) | 0.465 | 0.946 | 0.909 | 8.6 | 0.57 | 20 | 23 | 2 | 35 | <0.001 |
|  | SBR Ventral Striatum (C) | 0.742 (0.635–0.850) | 0.767 | 0.622 | 0.702 | 2.03 | 0.37 | 33 | 10 | 14 | 23 | <0.001 |
|  | SBR Putamen (ant.) (I) | 0.815 (0.720–0.909) | 0.791 | 0.811 | 0.829 | 4.18 | 0.26 | 34 | 9 | 7 | 30 | <0.001 |
|  | SBR Putamen (ant.) (C) | 0.827 (0.735–0.919) | 0.860 | 0.730 | 0.787 | 3.18 | 0.19 | 37 | 6 | 10 | 27 | <0.001 |
|  | SBR Putamen (post.) (I) | 0.832 (0.742–0.922) | 0.767 | 0.811 | 0.825 | 4.06 | 0.29 | 33 | 10 | 7 | 30 | <0.001 |
|  | SBR Putamen (post.) (C) | 0.855 (0.770–0.940) | 0.860 | 0.784 | 0.822 | 3.98 | 0.18 | 37 | 6 | 8 | 29 | <0.001 |
|  | SBR Caudate (I) | 0.672 (0.554–0.790) | 0.767 | 0.541 | 0.660 | 1.67 | 0.43 | 33 | 10 | 17 | 20 | 0.008 |
|  | SBR Caudate (C) | 0.708 (0.593–0.822) | 0.884 | 0.486 | 0.667 | 1.72 | 0.24 | 38 | 5 | 19 | 18 | 0.001 |
|  | Cau-Put (ant.) ratio | 0.790 (0.678–0.901) | 0.977 | 0.622 | 0.750 | 2.58 | 0.04 | 42 | 1 | 14 | 23 | <0.001 |
|  | Cau-Put (post.) ratio | 0.802 (0.698–0.906) | 0.767 | 0.811 | 0.825 | 4.06 | 0.29 | 33 | 10 | 7 | 30 | <0.001 |
|  | AI Ventral Striatum | 0.522 (0.393–0.651) | 0.349 | 0.784 | 0.652 | 1.61 | 0.83 | 15 | 28 | 8 | 29 | 0.739 |
|  | AI Putamen (ant.) | 0.569 (0.439–0.699) | 0.953 | 0.270 | 0.603 | 1.31 | 0.17 | 41 | 2 | 27 | 10 | 0.293 |
|  | AI Putamen (post.) | 0.465 (0.329–0.601) | 0.860 | 0.297 | 0.587 | 1.22 | 0.47 | 37 | 6 | 26 | 11 | 0.592 |
|  | AI Caudate | 0.481 (0.352–0.611) | 0.953 | 0.108 | 0.554 | 1.07 | 0.43 | 41 | 2 | 33 | 4 | 0.78 |
|  | SBR Cognitive (I) | 0.762 (0.657–0.867) | 0.814 | 0.649 | 0.729 | 2.32 | 0.29 | 35 | 8 | 13 | 24 | <0.001 |
|  | SBR Cognitive (C) | 0.789 (0.689–0.890) | 0.814 | 0.730 | 0.778 | 3.01 | 0.25 | 35 | 8 | 10 | 27 | <0.001 |
|  | SBR Executive (I) | 0.755 (0.650–0.861) | 0.767 | 0.676 | 0.733 | 2.37 | 0.34 | 33 | 10 | 12 | 25 | <0.001 |
|  | SBR Executive (C) | 0.782 (0.680–0.884) | 0.791 | 0.730 | 0.773 | 2.93 | 0.29 | 34 | 9 | 10 | 27 | <0.001 |
|  | SBR Limbic (I) | 0.775 (0.673–0.877) | 0.651 | 0.811 | 0.800 | 3.44 | 0.43 | 28 | 15 | 7 | 30 | <0.001 |
|  | SBR Limbic (C) | 0.791 (0.692–0.890) | 0.814 | 0.703 | 0.761 | 2.74 | 0.26 | 35 | 8 | 11 | 26 | <0.001 |
|  | SBR Sensorimotor (I) | 0.821 (0.729–0.914) | 0.744 | 0.811 | 0.821 | 3.93 | 0.32 | 32 | 11 | 7 | 30 | <0.001 |
|  | SBR Sensorimotor (C) | 0.839 (0.749–0.929) | 0.791 | 0.811 | 0.829 | 4.18 | 0.26 | 34 | 9 | 7 | 30 | <0.001 |
|  | Sen-Cog ratio | 0.768 (0.662–0.874) | 0.744 | 0.703 | 0.744 | 2.5 | 0.36 | 32 | 11 | 11 | 26 | <0.001 |
|  | Sen-Exe ratio | 0.758 (0.651–0.865) | 0.767 | 0.622 | 0.702 | 2.03 | 0.37 | 33 | 10 | 14 | 23 | <0.001 |
|  | Sen-Lim ratio | 0.760 (0.649–0.872) | 0.814 | 0.703 | 0.761 | 2.74 | 0.26 | 35 | 8 | 11 | 26 | <0.001 |
|  | AI Cognitive | 0.541 (0.411–0.672) | 0.837 | 0.324 | 0.590 | 1.24 | 0.5 | 36 | 7 | 25 | 12 | 0.527 |
|  | AI Executive | 0.563 (0.434–0.693) | 0.953 | 0.270 | 0.603 | 1.31 | 0.17 | 41 | 2 | 27 | 10 | 0.335 |
|  | AI Limbic | 0.588 (0.462–0.714) | 0.372 | 0.865 | 0.762 | 2.75 | 0.73 | 16 | 27 | 5 | 32 | 0.177 |
|  | AI Sensorimotor | 0.589 (0.460–0.718) | 0.814 | 0.432 | 0.625 | 1.43 | 0.43 | 35 | 8 | 21 | 16 | 0.174 |
| **CBD (n=43)**  **vs. MSA-C (n=12)** | SBR Ventral Striatum (I) | 0.700 (0.532–0.867) | 0.512 | 0.917 | 0.957 | 6.14 | 0.53 | 22 | 21 | 1 | 11 | 0.036 |
|  | SBR Ventral Striatum (C) | 0.603 (0.427–0.778) | 0.465 | 0.917 | 0.952 | 5.58 | 0.58 | 20 | 23 | 1 | 11 | 0.289 |
|  | SBR Putamen (ant.) (I) | 0.709 (0.548–0.870) | 0.558 | 0.917 | 0.960 | 6.7 | 0.48 | 24 | 19 | 1 | 11 | 0.027 |
|  | SBR Putamen (ant.) (C) | 0.578 (0.404–0.751) | 0.442 | 0.833 | 0.905 | 2.65 | 0.67 | 19 | 24 | 2 | 10 | 0.426 |
|  | SBR Putamen (post.) (I) | 0.738 (0.591–0.885) | 0.605 | 0.917 | 0.963 | 7.26 | 0.43 | 26 | 17 | 1 | 11 | 0.011 |
|  | SBR Putamen (post.) (C) | 0.649 (0.480–0.818) | 0.558 | 0.833 | 0.923 | 3.35 | 0.53 | 24 | 19 | 2 | 10 | 0.12 |
|  | SBR Caudate (I) | 0.771 (0.609–0.933) | 0.721 | 0.917 | 0.969 | 8.65 | 0.3 | 31 | 12 | 1 | 11 | 0.004 |
|  | SBR Caudate (C) | 0.702 (0.530–0.873) | 0.628 | 0.833 | 0.931 | 3.77 | 0.45 | 27 | 16 | 2 | 10 | 0.034 |
|  | Cau-Put (ant.) ratio | 0.815 (0.638–0.991) | 0.814 | 0.750 | 0.921 | 3.26 | 0.25 | 35 | 8 | 3 | 9 | <0.001 |
|  | Cau-Put (post.) ratio | 0.596 (0.424–0.768) | 0.558 | 0.750 | 0.889 | 2.23 | 0.59 | 24 | 19 | 3 | 9 | 0.318 |
|  | AI Ventral Striatum | 0.551 (0.385–0.717) | 0.233 | 1.000 | 1.000 |  | 0.77 | 10 | 33 | 0 | 12 | 0.596 |
|  | AI Putamen (ant.) | 0.635 (0.460–0.810) | 0.419 | 0.833 | 0.900 | 2.51 | 0.7 | 18 | 25 | 2 | 10 | 0.16 |
|  | AI Putamen (post.) | 0.832 (0.722–0.943) | 0.837 | 0.750 | 0.923 | 3.35 | 0.22 | 36 | 7 | 3 | 9 | <0.001 |
|  | AI Caudate | 0.787 (0.661–0.912) | 0.698 | 0.917 | 0.968 | 8.37 | 0.33 | 30 | 13 | 1 | 11 | 0.003 |
|  | SBR Cognitive (I) | 0.734 (0.574–0.895) | 0.698 | 0.833 | 0.938 | 4.19 | 0.36 | 30 | 13 | 2 | 10 | 0.013 |
|  | SBR Cognitive (C) | 0.651 (0.483–0.819) | 0.558 | 0.917 | 0.960 | 6.7 | 0.48 | 24 | 19 | 1 | 11 | 0.115 |
|  | SBR Executive (I) | 0.748 (0.584–0.912) | 0.651 | 0.917 | 0.966 | 7.81 | 0.38 | 28 | 15 | 1 | 11 | 0.008 |
|  | SBR Executive (C) | 0.667 (0.495–0.838) | 0.488 | 0.917 | 0.955 | 5.86 | 0.56 | 21 | 22 | 1 | 11 | 0.081 |
|  | SBR Limbic (I) | 0.684 (0.522–0.846) | 0.512 | 0.917 | 0.957 | 6.14 | 0.53 | 22 | 21 | 1 | 11 | 0.053 |
|  | SBR Limbic (C) | 0.597 (0.423–0.771) | 0.488 | 0.917 | 0.955 | 5.86 | 0.56 | 21 | 22 | 1 | 11 | 0.317 |
|  | SBR Sensorimotor (I) | 0.733 (0.577–0.888) | 0.605 | 0.917 | 0.963 | 7.26 | 0.43 | 26 | 17 | 1 | 11 | 0.013 |
|  | SBR Sensorimotor (C) | 0.688 (0.520–0.856) | 0.535 | 0.917 | 0.958 | 6.42 | 0.51 | 23 | 20 | 1 | 11 | 0.048 |
|  | Sen-Cog ratio | 0.569 (0.414–0.724) | 0.302 | 1.000 | 1.000 |  | 0.7 | 13 | 30 | 0 | 12 | 0.475 |
|  | Sen-Exe ratio | 0.535 (0.376–0.693) | 0.233 | 1.000 | 1.000 |  | 0.77 | 10 | 33 | 0 | 12 | 0.721 |
|  | Sen-Lim ratio | 0.659 (0.510–0.808) | 0.349 | 1.000 | 1.000 |  | 0.65 | 15 | 28 | 0 | 12 | 0.096 |
|  | AI Cognitive | 0.771 (0.646–0.897) | 0.535 | 1.000 | 1.000 |  | 0.47 | 23 | 20 | 0 | 12 | 0.004 |
|  | AI Executive | 0.745 (0.615–0.876) | 0.581 | 0.917 | 0.962 | 6.98 | 0.46 | 25 | 18 | 1 | 11 | 0.01 |
|  | AI Limbic | 0.661 (0.519–0.803) | 0.442 | 1.000 | 1.000 |  | 0.56 | 19 | 24 | 0 | 12 | 0.093 |
|  | AI Sensorimotor | 0.791 (0.673–0.908) | 0.581 | 1.000 | 1.000 |  | 0.42 | 25 | 18 | 0 | 12 | 0.002 |
| **CBD (n=43)**  **vs. PSP (n=127)** | SBR Ventral Striatum (I) | 0.606 (0.507–0.706) | 0.651 | 0.575 | 0.341 | 1.53 | 0.61 | 28 | 15 | 54 | 73 | 0.037 |
|  | SBR Ventral Striatum (C) | 0.622 (0.528–0.716) | 0.767 | 0.457 | 0.324 | 1.41 | 0.51 | 33 | 10 | 69 | 58 | 0.017 |
|  | SBR Putamen (ant.) (I) | 0.668 (0.576–0.761) | 0.721 | 0.559 | 0.356 | 1.63 | 0.5 | 31 | 12 | 56 | 71 | <0.001 |
|  | SBR Putamen (ant.) (C) | 0.698 (0.609–0.788) | 0.814 | 0.520 | 0.365 | 1.69 | 0.36 | 35 | 8 | 61 | 66 | <0.001 |
|  | SBR Putamen (post.) (I) | 0.680 (0.587–0.774) | 0.744 | 0.543 | 0.356 | 1.63 | 0.47 | 32 | 11 | 58 | 69 | <0.001 |
|  | SBR Putamen (post.) (C) | 0.729 (0.643–0.814) | 0.860 | 0.512 | 0.374 | 1.76 | 0.27 | 37 | 6 | 62 | 65 | <0.001 |
|  | SBR Caudate (I) | 0.615 (0.518–0.713) | 0.395 | 0.811 | 0.415 | 2.09 | 0.75 | 17 | 26 | 24 | 103 | 0.024 |
|  | SBR Caudate (C) | 0.668 (0.577–0.759) | 0.884 | 0.417 | 0.339 | 1.52 | 0.28 | 38 | 5 | 74 | 53 | 0.001 |
|  | Cau-Put (ant.) ratio | 0.544 (0.450–0.639) | 0.651 | 0.480 | 0.298 | 1.25 | 0.73 | 28 | 15 | 66 | 61 | 0.385 |
|  | Cau-Put (post.) ratio | 0.580 (0.485–0.676) | 0.791 | 0.417 | 0.315 | 1.36 | 0.5 | 34 | 9 | 74 | 53 | 0.115 |
|  | AI Ventral Striatum | 0.568 (0.460–0.675) | 0.326 | 0.866 | 0.452 | 2.43 | 0.78 | 14 | 29 | 17 | 110 | 0.186 |
|  | AI Putamen (ant.) | 0.515 (0.419–0.612) | 0.930 | 0.165 | 0.274 | 1.11 | 0.42 | 40 | 3 | 106 | 21 | 0.766 |
|  | AI Putamen (post.) | 0.606 (0.500–0.711) | 0.512 | 0.732 | 0.393 | 1.91 | 0.67 | 22 | 21 | 34 | 93 | 0.039 |
|  | AI Caudate | 0.562 (0.459–0.664) | 0.651 | 0.496 | 0.304 | 1.29 | 0.7 | 28 | 15 | 64 | 63 | 0.228 |
|  | SBR Cognitive (I) | 0.648 (0.552–0.743) | 0.651 | 0.630 | 0.373 | 1.76 | 0.55 | 28 | 15 | 47 | 80 | 0.004 |
|  | SBR Cognitive (C) | 0.686 (0.596–0.776) | 0.814 | 0.528 | 0.368 | 1.72 | 0.35 | 35 | 8 | 60 | 67 | <0.001 |
|  | SBR Executive (I) | 0.649 (0.555–0.743) | 0.488 | 0.748 | 0.396 | 1.94 | 0.68 | 21 | 22 | 32 | 95 | 0.004 |
|  | SBR Executive (C) | 0.690 (0.600–0.780) | 0.884 | 0.433 | 0.345 | 1.56 | 0.27 | 38 | 5 | 72 | 55 | <0.001 |
|  | SBR Limbic (I) | 0.630 (0.531–0.729) | 0.628 | 0.622 | 0.360 | 1.66 | 0.6 | 27 | 16 | 48 | 79 | 0.011 |
|  | SBR Limbic (C) | 0.664 (0.573–0.755) | 0.791 | 0.512 | 0.354 | 1.62 | 0.41 | 34 | 9 | 62 | 65 | 0.001 |
|  | SBR Sensorimotor (I) | 0.678 (0.585–0.772) | 0.767 | 0.535 | 0.359 | 1.65 | 0.43 | 33 | 10 | 59 | 68 | <0.001 |
|  | SBR Sensorimotor (C) | 0.723 (0.637–0.809) | 0.791 | 0.575 | 0.386 | 1.86 | 0.36 | 34 | 9 | 54 | 73 | <0.001 |
|  | Sen-Cog ratio | 0.638 (0.542–0.734) | 0.605 | 0.677 | 0.388 | 1.87 | 0.58 | 26 | 17 | 41 | 86 | 0.007 |
|  | Sen-Exe ratio | 0.592 (0.494–0.690) | 0.651 | 0.512 | 0.311 | 1.33 | 0.68 | 28 | 15 | 62 | 65 | 0.072 |
|  | Sen-Lim ratio | 0.674 (0.581–0.767) | 0.814 | 0.520 | 0.365 | 1.69 | 0.36 | 35 | 8 | 61 | 66 | <0.001 |
|  | AI Cognitive | 0.534 (0.427–0.641) | 0.395 | 0.764 | 0.362 | 1.67 | 0.79 | 17 | 26 | 30 | 97 | 0.511 |
|  | AI Executive | 0.547 (0.442–0.652) | 0.372 | 0.772 | 0.356 | 1.63 | 0.81 | 16 | 27 | 29 | 98 | 0.362 |
|  | AI Limbic | 0.522 (0.415–0.629) | 0.395 | 0.724 | 0.327 | 1.43 | 0.83 | 17 | 26 | 35 | 92 | 0.668 |
|  | AI Sensorimotor | 0.555 (0.448–0.662) | 0.419 | 0.732 | 0.346 | 1.56 | 0.79 | 18 | 25 | 34 | 93 | 0.282 |
| **MSA-P (n=37)**  **vs. MSA-C (n=12)** | SBR Ventral Striatum (I) | 0.894 (0.748–1.000) | 0.838 | 0.917 | 0.969 | 10.05 | 0.18 | 31 | 6 | 1 | 11 | <0.001 |
|  | SBR Ventral Striatum (C) | 0.842 (0.694–0.990) | 0.811 | 0.917 | 0.968 | 9.73 | 0.21 | 30 | 7 | 1 | 11 | <0.001 |
|  | SBR Putamen (ant.) (I) | 0.939 (0.848–1.000) | 0.919 | 0.917 | 0.971 | 11.03 | 0.09 | 34 | 3 | 1 | 11 | <0.001 |
|  | SBR Putamen (ant.) (C) | 0.910 (0.826–0.994) | 0.811 | 0.917 | 0.968 | 9.73 | 0.21 | 30 | 7 | 1 | 11 | <0.001 |
|  | SBR Putamen (post.) (I) | 0.968 (0.925–1.000) | 0.892 | 0.917 | 0.971 | 10.7 | 0.12 | 33 | 4 | 1 | 11 | <0.001 |
|  | SBR Putamen (post.) (C) | 0.935 (0.858–1.000) | 0.865 | 0.917 | 0.970 | 10.38 | 0.15 | 32 | 5 | 1 | 11 | <0.001 |
|  | SBR Caudate (I) | 0.908 (0.766–1.000) | 0.973 | 0.833 | 0.947 | 5.84 | 0.03 | 36 | 1 | 2 | 10 | <0.001 |
|  | SBR Caudate (C) | 0.849 (0.698–1.000) | 0.757 | 0.917 | 0.966 | 9.08 | 0.27 | 28 | 9 | 1 | 11 | <0.001 |
|  | Cau-Put (ant.) ratio | 0.628 (0.472–0.785) | 0.432 | 1.000 | 1.000 |  | 0.57 | 16 | 21 | 0 | 12 | 0.188 |
|  | Cau-Put (post.) ratio | 0.831 (0.719–0.943) | 0.703 | 1.000 | 1.000 |  | 0.3 | 26 | 11 | 0 | 12 | <0.001 |
|  | AI Ventral Striatum | 0.600 (0.427–0.773) | 0.486 | 0.750 | 0.857 | 1.95 | 0.68 | 18 | 19 | 3 | 9 | 0.306 |
|  | AI Putamen (ant.) | 0.659 (0.497–0.820) | 0.514 | 0.833 | 0.905 | 3.08 | 0.58 | 19 | 18 | 2 | 10 | 0.104 |
|  | AI Putamen (post.) | 0.740 (0.605–0.875) | 0.622 | 0.917 | 0.958 | 7.46 | 0.41 | 23 | 14 | 1 | 11 | 0.014 |
|  | AI Caudate | 0.791 (0.662–0.919) | 0.703 | 0.917 | 0.963 | 8.43 | 0.32 | 26 | 11 | 1 | 11 | 0.003 |
|  | SBR Cognitive (I) | 0.928 (0.804–1.000) | 1.000 | 0.833 | 0.949 | 6 | 0 | 37 | 0 | 2 | 10 | <0.001 |
|  | SBR Cognitive (C) | 0.867 (0.733–1.000) | 0.838 | 0.917 | 0.969 | 10.05 | 0.18 | 31 | 6 | 1 | 11 | <0.001 |
|  | SBR Executive (I) | 0.930 (0.806–1.000) | 0.919 | 0.917 | 0.971 | 11.03 | 0.09 | 34 | 3 | 1 | 11 | <0.001 |
|  | SBR Executive (C) | 0.874 (0.744–1.000) | 0.811 | 0.917 | 0.968 | 9.73 | 0.21 | 30 | 7 | 1 | 11 | <0.001 |
|  | SBR Limbic (I) | 0.923 (0.814–1.000) | 0.892 | 0.917 | 0.971 | 10.7 | 0.12 | 33 | 4 | 1 | 11 | <0.001 |
|  | SBR Limbic (C) | 0.847 (0.706–0.988) | 0.838 | 0.917 | 0.969 | 10.05 | 0.18 | 31 | 6 | 1 | 11 | <0.001 |
|  | SBR Sensorimotor (I) | 0.964 (0.907–1.000) | 0.919 | 0.917 | 0.971 | 11.03 | 0.09 | 34 | 3 | 1 | 11 | <0.001 |
|  | SBR Sensorimotor (C) | 0.926 (0.834–1.000) | 0.919 | 0.917 | 0.971 | 11.03 | 0.09 | 34 | 3 | 1 | 11 | <0.001 |
|  | Sen-Cog ratio | 0.882 (0.789–0.974) | 0.703 | 1.000 | 1.000 |  | 0.3 | 26 | 11 | 0 | 12 | <0.001 |
|  | Sen-Exe ratio | 0.838 (0.726–0.950) | 0.622 | 1.000 | 1.000 |  | 0.38 | 23 | 14 | 0 | 12 | <0.001 |
|  | Sen-Lim ratio | 0.902 (0.818–0.986) | 0.784 | 1.000 | 1.000 |  | 0.22 | 29 | 8 | 0 | 12 | <0.001 |
|  | AI Cognitive | 0.769 (0.640–0.898) | 0.649 | 1.000 | 1.000 |  | 0.35 | 24 | 13 | 0 | 12 | 0.006 |
|  | AI Executive | 0.765 (0.633–0.896) | 0.649 | 0.917 | 0.960 | 7.78 | 0.38 | 24 | 13 | 1 | 11 | 0.007 |
|  | AI Limbic | 0.825 (0.707–0.943) | 0.622 | 1.000 | 1.000 |  | 0.38 | 23 | 14 | 0 | 12 | <0.001 |
|  | AI Sensorimotor | 0.811 (0.693–0.929) | 0.622 | 1.000 | 1.000 |  | 0.38 | 23 | 14 | 0 | 12 | 0.001 |
| **MSA-P (n=37)**  **vs. PSP (n=127)** | SBR Ventral Striatum (I) | 0.632 (0.537–0.727) | 0.973 | 0.268 | 0.279 | 1.33 | 0.1 | 36 | 1 | 93 | 34 | 0.015 |
|  | SBR Ventral Striatum (C) | 0.615 (0.517–0.712) | 0.459 | 0.740 | 0.340 | 1.77 | 0.73 | 17 | 20 | 33 | 94 | 0.034 |
|  | SBR Putamen (ant.) (I) | 0.658 (0.563–0.754) | 0.676 | 0.685 | 0.385 | 2.15 | 0.47 | 25 | 12 | 40 | 87 | 0.003 |
|  | SBR Putamen (ant.) (C) | 0.638 (0.543–0.733) | 0.730 | 0.551 | 0.321 | 1.63 | 0.49 | 27 | 10 | 57 | 70 | 0.011 |
|  | SBR Putamen (post.) (I) | 0.690 (0.595–0.786) | 0.703 | 0.677 | 0.388 | 2.18 | 0.44 | 26 | 11 | 41 | 86 | <0.001 |
|  | SBR Putamen (post.) (C) | 0.640 (0.544–0.736) | 0.784 | 0.504 | 0.315 | 1.58 | 0.43 | 29 | 8 | 63 | 64 | 0.01 |
|  | SBR Caudate (I) | 0.557 (0.454–0.659) | 0.622 | 0.543 | 0.284 | 1.36 | 0.7 | 23 | 14 | 58 | 69 | 0.295 |
|  | SBR Caudate (C) | 0.523 (0.419–0.627) | 0.568 | 0.559 | 0.273 | 1.29 | 0.77 | 21 | 16 | 56 | 71 | 0.671 |
|  | Cau-Put (ant.) ratio | 0.742 (0.639–0.845) | 0.676 | 0.819 | 0.521 | 3.73 | 0.4 | 25 | 12 | 23 | 104 | <0.001 |
|  | Cau-Put (post.) ratio | 0.730 (0.634–0.825) | 0.865 | 0.528 | 0.348 | 1.83 | 0.26 | 32 | 5 | 60 | 67 | <0.001 |
|  | AI Ventral Striatum | 0.537 (0.429–0.645) | 0.838 | 0.283 | 0.254 | 1.17 | 0.57 | 31 | 6 | 91 | 36 | 0.5 |
|  | AI Putamen (ant.) | 0.551 (0.437–0.665) | 0.243 | 0.937 | 0.529 | 3.86 | 0.81 | 9 | 28 | 8 | 119 | 0.347 |
|  | AI Putamen (post.) | 0.604 (0.472–0.735) | 0.432 | 0.953 | 0.727 | 9.15 | 0.6 | 16 | 21 | 6 | 121 | 0.056 |
|  | AI Caudate | 0.582 (0.473–0.691) | 0.703 | 0.480 | 0.283 | 1.35 | 0.62 | 26 | 11 | 66 | 61 | 0.129 |
|  | SBR Cognitive (I) | 0.627 (0.529–0.725) | 0.649 | 0.654 | 0.353 | 1.87 | 0.54 | 24 | 13 | 44 | 83 | 0.019 |
|  | SBR Cognitive (C) | 0.598 (0.499–0.697) | 0.649 | 0.575 | 0.308 | 1.53 | 0.61 | 24 | 13 | 54 | 73 | 0.07 |
|  | SBR Executive (I) | 0.615 (0.516–0.714) | 0.649 | 0.606 | 0.324 | 1.65 | 0.58 | 24 | 13 | 50 | 77 | 0.033 |
|  | SBR Executive (C) | 0.582 (0.483–0.682) | 0.730 | 0.496 | 0.297 | 1.45 | 0.54 | 27 | 10 | 64 | 63 | 0.128 |
|  | SBR Limbic (I) | 0.654 (0.559–0.749) | 0.622 | 0.661 | 0.348 | 1.84 | 0.57 | 23 | 14 | 43 | 84 | 0.004 |
|  | SBR Limbic (C) | 0.629 (0.532–0.726) | 0.649 | 0.622 | 0.333 | 1.72 | 0.56 | 24 | 13 | 48 | 79 | 0.017 |
|  | SBR Sensorimotor (I) | 0.656 (0.560–0.752) | 0.676 | 0.669 | 0.373 | 2.04 | 0.48 | 25 | 12 | 42 | 85 | 0.004 |
|  | SBR Sensorimotor (C) | 0.615 (0.518–0.713) | 0.757 | 0.528 | 0.318 | 1.6 | 0.46 | 28 | 9 | 60 | 67 | 0.033 |
|  | Sen-Cog ratio | 0.648 (0.548–0.747) | 0.703 | 0.551 | 0.313 | 1.57 | 0.54 | 26 | 11 | 57 | 70 | 0.006 |
|  | Sen-Exe ratio | 0.680 (0.580–0.780) | 0.541 | 0.756 | 0.392 | 2.21 | 0.61 | 20 | 17 | 31 | 96 | <0.001 |
|  | Sen-Lim ratio | 0.574 (0.474–0.674) | 0.784 | 0.441 | 0.290 | 1.4 | 0.49 | 29 | 8 | 71 | 56 | 0.171 |
|  | AI Cognitive | 0.572 (0.451–0.692) | 0.324 | 0.890 | 0.462 | 2.94 | 0.76 | 12 | 25 | 14 | 113 | 0.187 |
|  | AI Executive | 0.594 (0.481–0.708) | 0.324 | 0.906 | 0.500 | 3.43 | 0.75 | 12 | 25 | 12 | 115 | 0.082 |
|  | AI Limbic | 0.572 (0.469–0.675) | 0.649 | 0.496 | 0.273 | 1.29 | 0.71 | 24 | 13 | 64 | 63 | 0.182 |
|  | AI Sensorimotor | 0.637 (0.515–0.758) | 0.541 | 0.795 | 0.435 | 2.64 | 0.58 | 20 | 17 | 26 | 101 | 0.011 |
| **MSA-C (n=12)**  **vs. PSP (n=127)** | SBR Ventral Striatum (I) | 0.780 (0.635–0.925) | 0.917 | 0.646 | 0.196 | 2.59 | 0.13 | 11 | 1 | 45 | 82 | 0.001 |
|  | SBR Ventral Striatum (C) | 0.717 (0.578–0.856) | 0.917 | 0.630 | 0.190 | 2.48 | 0.13 | 11 | 1 | 47 | 80 | 0.013 |
|  | SBR Putamen (ant.) (I) | 0.844 (0.733–0.956) | 0.917 | 0.780 | 0.282 | 4.16 | 0.11 | 11 | 1 | 28 | 99 | <0.001 |
|  | SBR Putamen (ant.) (C) | 0.782 (0.674–0.890) | 0.833 | 0.717 | 0.217 | 2.94 | 0.23 | 10 | 2 | 36 | 91 | 0.001 |
|  | SBR Putamen (post.) (I) | 0.895 (0.806–0.984) | 0.833 | 0.906 | 0.455 | 8.82 | 0.18 | 10 | 2 | 12 | 115 | <0.001 |
|  | SBR Putamen (post.) (C) | 0.855 (0.750–0.960) | 0.833 | 0.827 | 0.312 | 4.81 | 0.2 | 10 | 2 | 22 | 105 | <0.001 |
|  | SBR Caudate (I) | 0.860 (0.724–0.995) | 0.917 | 0.827 | 0.333 | 5.29 | 0.1 | 11 | 1 | 22 | 105 | <0.001 |
|  | SBR Caudate (C) | 0.827 (0.681–0.972) | 0.833 | 0.803 | 0.286 | 4.23 | 0.21 | 10 | 2 | 25 | 102 | <0.001 |
|  | Cau-Put (ant.) ratio | 0.736 (0.576–0.895) | 0.667 | 0.819 | 0.258 | 3.68 | 0.41 | 8 | 4 | 23 | 104 | 0.007 |
|  | Cau-Put (post.) ratio | 0.467 (0.342–0.591) | 0.750 | 0.472 | 0.118 | 1.42 | 0.53 | 9 | 3 | 67 | 60 | 0.708 |
|  | AI Ventral Striatum | 0.646 (0.495–0.798) | 0.667 | 0.606 | 0.138 | 1.69 | 0.55 | 8 | 4 | 50 | 77 | 0.095 |
|  | AI Putamen (ant.) | 0.624 (0.468–0.780) | 0.833 | 0.441 | 0.123 | 1.49 | 0.38 | 10 | 2 | 71 | 56 | 0.157 |
|  | AI Putamen (post.) | 0.791 (0.683–0.899) | 0.833 | 0.693 | 0.204 | 2.71 | 0.24 | 10 | 2 | 39 | 88 | <0.001 |
|  | AI Caudate | 0.733 (0.608–0.859) | 0.917 | 0.559 | 0.164 | 2.08 | 0.15 | 11 | 1 | 56 | 71 | 0.008 |
|  | SBR Cognitive (I) | 0.854 (0.729–0.978) | 0.833 | 0.882 | 0.400 | 7.06 | 0.19 | 10 | 2 | 15 | 112 | <0.001 |
|  | SBR Cognitive (C) | 0.801 (0.673–0.929) | 0.917 | 0.693 | 0.220 | 2.99 | 0.12 | 11 | 1 | 39 | 88 | <0.001 |
|  | SBR Executive (I) | 0.859 (0.732–0.986) | 0.917 | 0.819 | 0.324 | 5.06 | 0.1 | 11 | 1 | 23 | 104 | <0.001 |
|  | SBR Executive (C) | 0.815 (0.688–0.942) | 0.917 | 0.693 | 0.220 | 2.99 | 0.12 | 11 | 1 | 39 | 88 | <0.001 |
|  | SBR Limbic (I) | 0.813 (0.688–0.938) | 0.917 | 0.732 | 0.244 | 3.42 | 0.11 | 11 | 1 | 34 | 93 | <0.001 |
|  | SBR Limbic (C) | 0.746 (0.618–0.874) | 0.917 | 0.677 | 0.212 | 2.84 | 0.12 | 11 | 1 | 41 | 86 | 0.005 |
|  | SBR Sensorimotor (I) | 0.888 (0.782–0.993) | 0.917 | 0.858 | 0.379 | 6.47 | 0.1 | 11 | 1 | 18 | 109 | <0.001 |
|  | SBR Sensorimotor (C) | 0.864 (0.759–0.970) | 0.917 | 0.795 | 0.297 | 4.48 | 0.1 | 11 | 1 | 26 | 101 | <0.001 |
|  | Sen-Cog ratio | 0.741 (0.645–0.837) | 0.917 | 0.598 | 0.177 | 2.28 | 0.14 | 11 | 1 | 51 | 76 | 0.006 |
|  | Sen-Exe ratio | 0.652 (0.534–0.769) | 1.000 | 0.354 | 0.128 | 1.55 | 0 | 12 | 0 | 82 | 45 | 0.084 |
|  | Sen-Lim ratio | 0.819 (0.741–0.897) | 1.000 | 0.638 | 0.207 | 2.76 | 0 | 12 | 0 | 46 | 81 | <0.001 |
|  | AI Cognitive | 0.781 (0.681–0.882) | 1.000 | 0.535 | 0.169 | 2.15 | 0 | 12 | 0 | 59 | 68 | 0.001 |
|  | AI Executive | 0.720 (0.604–0.836) | 0.917 | 0.528 | 0.155 | 1.94 | 0.16 | 11 | 1 | 60 | 67 | 0.012 |
|  | AI Limbic | 0.721 (0.615–0.827) | 1.000 | 0.488 | 0.156 | 1.95 | 0 | 12 | 0 | 65 | 62 | 0.011 |
|  | AI Sensorimotor | 0.784 (0.675–0.893) | 0.917 | 0.559 | 0.164 | 2.08 | 0.15 | 11 | 1 | 56 | 71 | 0.001 |
| For each pairwise comparison, the first-named group was treated as the positive class. The ROC direction was determined automatically to ensure AUC ≥ 0.5. Optimal cut-offs were selected using the Youden index (J = sensitivity + specificity − 1). AUC 95% confidence intervals and p-values were computed using the DeLong method. Sensitivity and specificity were verified directly from classification at the reported cut-off. Positive predictive value (PPV) is reported positive and negative likelihood ratios (LR+ and LR−) as prevalence-independent measures of diagnostic test performance: LR+ = sensitivity / (1 − specificity); LR− = (1 − sensitivity) / specificity. Comparisons involving MSA-C (n = 12) should be interpreted with caution due to the very small sample size, which produces unstable point estimates for sensitivity, specificity, and likelihood ratios.  Abbreviations: AUC = area under the curve; CBD = corticobasal degeneration; CI = confidence interval; FN = false negative; FP = false positive; LR+ = positive likelihood ratio; LR− = negative likelihood ratio; MSA-P = multiple system atrophy parkinsonian type; MSA-C = multiple system atrophy cerebellar type; PPV = positive predictive value; PSP = progressive supranuclear palsy; TN = true negative; TP = true positive. | | | | | | | | | | | | |

**Figure S1.** Variable importance ranking of [¹²³I]FP-CIT SPECT semiquantitative metrics based on the mean decrease in Gini impurity, averaged across all 100 cross-validation folds. Higher values indicate greater contribution to the overall classification performance of the Random Forest model.

| **Table S4. Cohort characteristics (MSA-P and MSA-C combined as MSA)** | | | | | |
| --- | --- | --- | --- | --- | --- |
|  | **CBD** | **MSA** | **PSP** | **PD** | **p*** |
| **n** | 43 | 49 | 127 | 487 |  |
| **Sex, n (%) Female** | 18 (42%) | 23 (47%) | 56 (44%) | 177 (36%) | 0.227 |
| **Age** | 72.1 (6.5) | 62.1 (8.2) | 71.8 (7.1) | 60.7 (10.6) | <0.001 |
| **Disease duration** | 2.7 (2.0) | 2.3 (1.8) | 2.4 (1.9) | 5.6 (6.2) | <0.001 |
| **SBR Ventral Striatum (I)** | 1.4 (0.5) | 1.2 (0.5) | 1.2 (0.5) | 1.4 (0.5) | <0.001 |
| **SBR Ventral Striatum (C)** | 1.5 (0.4) | 1.2 (0.5) | 1.3 (0.5) | 1.5 (0.5) | <0.001 |
| **SBR Putamen (ant.) (I)** | 1.9 (0.6) | 1.4 (0.7) | 1.5 (0.6) | 1.6 (0.7) | 0.005 |
| **SBR Putamen (ant.) (C)** | 2.1 (0.6) | 1.5 (0.7) | 1.6 (0.6) | 1.8 (0.7) | <0.001 |
| **SBR Putamen (post.) (I)** | 1.3 (0.5) | 0.9 (0.6) | 1.0 (0.4) | 0.9 (0.4) | <0.001 |
| **SBR Putamen (post.) (C)** | 1.6 (0.5) | 1.1 (0.6) | 1.1 (0.5) | 1.1 (0.5) | <0.001 |
| **SBR Caudate (I)** | 1.2 (0.5) | 1.1 (0.5) | 1.0 (0.4) | 1.2 (0.5) | <0.001 |
| **SBR Caudate (C)** | 1.4 (0.4) | 1.2 (0.5) | 1.1 (0.5) | 1.3 (0.5) | <0.001 |
| **SBR Cognitive (I)** | 1.5 (0.5) | 1.2 (0.6) | 1.2 (0.5) | 1.4 (0.6) | 0.003 |
| **SBR Cognitive (C)** | 1.7 (0.5) | 1.3 (0.6) | 1.3 (0.5) | 1.5 (0.6) | <0.001 |
| **SBR Executive (I)** | 1.5 (0.5) | 1.3 (0.6) | 1.2 (0.5) | 1.4 (0.6) | 0.003 |
| **SBR Executive (C)** | 1.7 (0.5) | 1.4 (0.6) | 1.3 (0.6) | 1.6 (0.6) | <0.001 |
| **SBR Limbic (I)** | 1.5 (0.5) | 1.2 (0.6) | 1.3 (0.5) | 1.4 (0.5) | 0.004 |
| **SBR Limbic (C)** | 1.7 (0.5) | 1.3 (0.5) | 1.4 (0.5) | 1.5 (0.6) | <0.001 |
| **SBR Sensorimotor (I)** | 1.3 (0.5) | 1.0 (0.5) | 1.0 (0.4) | 1.0 (0.4) | <0.001 |
| **SBR Sensorimotor (C)** | 1.5 (0.5) | 1.1 (0.6) | 1.1 (0.5) | 1.2 (0.5) | <0.001 |
| **Cau-Put (ant.) ratio** | 0.7 [0.5–1.0] | 0.8 [0.3–1.0] | 0.7 [0.2–1.2] | 0.8 [0.3–1.9] | <0.001 |
| **Cau-Put (post.) ratio** | 0.9 [0.5–4.3] | 1.1 [0.4–2.0] | 0.9 [0.4–2.1] | 1.3 [0.5–3.2] | <0.001 |
| **Sen-Cog ratio** | 0.9 [0.2–1.2] | 0.8 [0.6–1.5] | 0.8 [0.5–1.4] | 0.7 [0.5–2.2] | <0.001 |
| **Sen-Exe ratio** | 0.9 [0.3–1.2] | 0.8 [0.6–1.7] | 0.8 [0.5–1.2] | 0.7 [0.5–1.8] | <0.001 |
| **Sen-Lim ratio** | 0.9 [0.2–1.4] | 0.8 [0.4–1.2] | 0.8 [0.4–2.5] | 0.7 [0.4–4.6] | <0.001 |
| **AI Ventral Striatum** | 8.0 [0.8–64.8] | 9.4 [0.1–33.9] | 10.3 [0.1–91.3] | 10.7 [0.0–71.9] | 0.469 |
| **AI Putamen (ant.)** | 12.6 [0.2–35.2] | 12.1 [0.2–82.3] | 13.1 [0.1–97.5] | 14.4 [0.1–107.7] | 0.218 |
| **AI Putamen (post.)** | 21.1 [0.7–57.5] | 12.6 [0.3–86.7] | 13.6 [0.0–41.3] | 19.7 [0.0–61.6] | 0.002 |
| **AI Caudate** | 16.7 [0.2–77.1] | 12.4 [0.0–142.1] | 11.7 [0.0–138.3] | 13.1 [0.3–92.3] | 0.634 |
| **AI Cognitive** | 12.3 [0.1–47.4] | 9.7 [0.1–76.7] | 10.3 [0.1–46.9] | 12.6 [0.2–74.4] | 0.39 |
| **AI Executive** | 13.0 [0.1–46.4] | 12.1 [0.3–106.9] | 11.4 [0.0–70.2] | 13.7 [0.1–85.9] | 0.444 |
| **AI Limbic** | 8.4 [0.1–49.5] | 9.1 [0.5–50.7] | 9.1 [0.1–88.7] | 11.2 [0.0–77.7] | 0.408 |
| **AI Sensorimotor** | 17.7 [0.2–83.2] | 14.6 [0.5–77.6] | 14.7 [2.5–43.2] | 17.6 [0.0–92.7] | 0.471 |
| P-values for group comparisons were obtained using ANOVA for normally distributed continuous variables and the Kruskal-Wallis test for non-normally distributed variables. The chi-square test was used for sex.  Abbreviations: AI = asymmetry index; (ant.) = anterior; (C) = contralateral hemisphere relative to the most affected side; CBD = corticobasal degeneration; (I) = ipsilateral hemisphere relative to the most affected side; MSA = multiple system atrophy; PD = Parkinson's disease; PSP = progressive supranuclear palsy; SBR = standard binding ratio; (post.) = posterior. | | | | | |

**Table S5. Pair-wise comparisons of regional FP-CIT SPECT metrics for MSA (MSA-P and MSA-C combined as MSA)**

**against Parkinson’s disease**

|  | | | |
| --- | --- | --- | --- |
|  | **MSA vs. PD** | | |
|  | **β (CI)** | **p** | **d** |
| ***Anatomical striatal ROIs*** |  |  |  |
| SBR Ventral Striatum (I) | -0.3 (-0.5–-0.1) | **<0.001** | **0.6** |
| SBR Ventral Striatum (C) | -0.4 (-0.5–-0.2) | **<0.001** | **0.7** |
| SBR Putamen (ant.) (I) | -0.3 (-0.5–-0.1) | **0.005** | **0.5** |
| SBR Putamen (ant.) (C) | -0.4 (-0.6–-0.2) | **<0.001** | **0.7** |
| SBR Putamen (post.) (I) | -0.1 (-0.2–0.1) | 0.641 | 0.2 |
| SBR Putamen (post.) (C) | -0.1 (-0.3–0.1) | 0.17 | 0.3 |
| SBR Caudate (I) | -0.2 (-0.4–-0.1) | **0.004** | **0.6** |
| SBR Caudate (C) | -0.3 (-0.4–-0.1) | **<0.001** | **0.6** |
| Cau-Put (ant.) ratio | 1 (0.9–1) | 0.506 | 0.1 |
| Cau-Put (post.) ratio | 0.9 (0.8–1) | **0.003** | **0.5** |
| AI Ventral Striatum | 0.9 (0.7–1.2) | 0.507 | 0.1 |
| AI Putamen (ant.) | 1 (0.7–1.3) | 0.803 | 0 |
| AI Putamen (post.) | 1 (0.8–1.3) | 0.864 | 0.1 |
| AI Caudate | 1.3 (1–1.7) | 0.092 | 0.3 |
| ***Functional striatal ROIs*** |  |  |  |
| SBR Cognitive (I) | -0.3 (-0.5–-0.1) | **0.003** | **0.5** |
| SBR Cognitive (C) | -0.4 (-0.5–-0.2) | **<0.001** | **0.7** |
| SBR Executive (I) | -0.3 (-0.5–-0.1) | **0.007** | **0.5** |
| SBR Executive (C) | -0.4 (-0.5–-0.2) | **<0.001** | **0.6** |
| SBR Limbic (I) | -0.3 (-0.5–-0.1) | **<0.001** | **0.6** |
| SBR Limbic (C) | -0.4 (-0.5–-0.2) | **<0.001** | **0.7** |
| SBR Sensorimotor (I) | -0.1 (-0.3–0) | 0.148 | 0.3 |
| SBR Sensorimotor (C) | -0.2 (-0.3–0) | 0.066 | 0.3 |
| Sen-Cog ratio | 1.1 (1–1.1) | **<0.001** | **0.5** |
| Sen-Exe ratio | 1.1 (1–1.2) | **<0.001** | **0.6** |
| Sen-Lim ratio | 1.1 (1–1.2) | 0.077 | 0.3 |
| AI Cognitive | 1 (0.8–1.3) | 0.963 | 0 |
| AI Executive | 1.1 (0.9–1.4) | 0.635 | 0.1 |
| AI Limbic | 1 (0.8–1.3) | 0.893 | 0 |
| AI Sensorimotor | 1.2 (0.9–1.5) | 0.199 | 0.2 |
|  | | | |

Values represent standardized regression coefficients (β) with 95% confidence intervals (CI), *p*-values, and Cohen’s *d* effect sizes for pairwise group comparisons of FP-CIT specific binding ratios (SBRs), ratios, and asymmetry indices (AI) in anatomical and functional striatal regions of interest (ROIs). Comparisons were adjusted for age, sex, and disease duration. For SBR metrics, β denotes the absolute mean difference between each APS group and the PD group, adjusted for age, sex, and disease duration. For ratio and asymmetry index (AI) variables, generalized linear models with a Gamma distribution and log-link function were fitted. In these cases, the reported β indicates the ratio of means, defined as the mean value in the APS group divided by the mean value in the PD group. Values less than 1 indicate lower values in the APS group compared to PD, values greater than 1 indicate higher values, and values near 1 suggest no meaningful difference. Confidence intervals are reported on the same scale.

Abbreviations: AI = asymmetry index; (ant.) = anterior; (C) = contralateral hemisphere relative to the most affected side;; (I) = ipsilateral hemisphere relative to the most affected side; MSA = multiple system atrophy; PD = Parkinson’s disease; PSP = progressive supranuclear palsy; SBR = standard binding ratio; (post) = posterior.

| **Table S6. Diagnostic performance of FP-CIT metrics for distinguishing MSA (MSA-P and MSA-C combined as MSA) from PD** | | | | | | | | |
| --- | --- | --- | --- | --- | --- | --- | --- | --- |
|  |  | **AUC (95% CI)** | **Sensitivity** | **Specificity** | **PPV** | **LR+** | **LR−** | **p** |
| **MSA vs. PD** | SBR Ventral Striatum (I) | 0.622 (0.538–0.706) | 0.449 | 0.745 | 0.151 | 1.76 | 0.74 | 0.005 |
|  | SBR Ventral Striatum (C) | 0.636 (0.559–0.714) | 0.918 | 0.314 | 0.119 | 1.34 | 0.26 | 0.002 |
|  | SBR Putamen (ant.) (I) | 0.569 (0.483–0.656) | 0.531 | 0.692 | 0.148 | 1.72 | 0.68 | 0.11 |
|  | SBR Putamen (ant.) (C) | 0.597 (0.518–0.676) | 0.571 | 0.618 | 0.131 | 1.50 | 0.69 | 0.026 |
|  | SBR Putamen (post.) (I) | 0.524 (0.422–0.626) | 0.531 | 0.626 | 0.125 | 1.42 | 0.75 | 0.576 |
|  | SBR Putamen (post.) (C) | 0.517 (0.423–0.611) | 0.510 | 0.604 | 0.115 | 1.29 | 0.81 | 0.688 |
|  | SBR Caudate (I) | 0.587 (0.497–0.678) | 0.327 | 0.867 | 0.198 | 2.45 | 0.78 | 0.044 |
|  | SBR Caudate (C) | 0.589 (0.505–0.672) | 0.429 | 0.729 | 0.137 | 1.58 | 0.78 | 0.041 |
|  | SBR Cognitive (I) | 0.586 (0.497–0.675) | 0.510 | 0.725 | 0.157 | 1.85 | 0.68 | 0.047 |
|  | SBR Cognitive (C) | 0.602 (0.520–0.683) | 0.510 | 0.700 | 0.146 | 1.70 | 0.70 | 0.019 |
|  | SBR Executive (I) | 0.578 (0.487–0.668) | 0.490 | 0.715 | 0.147 | 1.72 | 0.71 | 0.073 |
|  | SBR Executive (C) | 0.592 (0.509–0.675) | 0.469 | 0.710 | 0.140 | 1.62 | 0.75 | 0.034 |
|  | SBR Limbic (I) | 0.604 (0.518–0.690) | 0.510 | 0.698 | 0.145 | 1.69 | 0.70 | 0.016 |
|  | SBR Limbic (C) | 0.624 (0.546–0.702) | 0.857 | 0.372 | 0.121 | 1.36 | 0.38 | 0.004 |
|  | SBR Sensorimotor (I) | 0.533 (0.434–0.631) | 0.551 | 0.632 | 0.131 | 1.50 | 0.71 | 0.452 |
|  | SBR Sensorimotor (C) | 0.527 (0.435–0.618) | 0.510 | 0.626 | 0.121 | 1.37 | 0.78 | 0.541 |
|  | Cau-Put (ant.) ratio | 0.532 (0.447–0.618) | 0.122 | 0.969 | 0.286 | 3.98 | 0.91 | 0.455 |
|  | Cau-Put (post.) ratio | 0.661 (0.570–0.751) | 0.531 | 0.809 | 0.218 | 2.78 | 0.58 | <0.001 |
|  | Sen-Cog ratio | 0.667 (0.582–0.752) | 0.429 | 0.871 | 0.250 | 3.31 | 0.66 | <0.001 |
|  | Sen-Exe ratio | 0.666 (0.585–0.747) | 0.510 | 0.782 | 0.191 | 2.34 | 0.63 | <0.001 |
|  | Sen-Lim ratio | 0.655 (0.567–0.743) | 0.408 | 0.875 | 0.247 | 3.26 | 0.68 | <0.001 |
|  | AI Ventral Striatum | 0.540 (0.458–0.622) | 0.857 | 0.257 | 0.104 | 1.15 | 0.56 | 0.357 |
|  | AI Putamen (ant.) | 0.536 (0.446–0.627) | 0.469 | 0.641 | 0.116 | 1.31 | 0.83 | 0.405 |
|  | AI Putamen (post.) | 0.551 (0.445–0.657) | 0.612 | 0.581 | 0.128 | 1.46 | 0.67 | 0.241 |
|  | AI Caudate | 0.510 (0.415–0.604) | 0.163 | 0.940 | 0.216 | 2.74 | 0.89 | 0.821 |
|  | AI Cognitive | 0.547 (0.450–0.644) | 0.184 | 0.957 | 0.300 | 4.26 | 0.85 | 0.278 |
|  | AI Executive | 0.519 (0.427–0.611) | 0.388 | 0.729 | 0.126 | 1.43 | 0.84 | 0.657 |
|  | AI Limbic | 0.539 (0.454–0.625) | 0.735 | 0.444 | 0.117 | 1.32 | 0.60 | 0.365 |
|  | AI Sensorimotor | 0.491 (0.394–0.588) | 0.347 | 0.768 | 0.131 | 1.50 | 0.85 | 0.837 |

Each atypical parkinsonian syndrome (APS) group was treated as the positive class in all comparisons. The ROC direction was determined automatically to ensure AUC ≥ 0.5. Optimal cut-offs were selected using the Youden index (J = sensitivity + specificity − 1). AUC 95% confidence intervals and p-values were computed using the DeLong method. Sensitivity and specificity were verified directly from classification at the reported cut-off. Positive predictive value (PPV) is reported positive and negative likelihood ratios (LR+ and LR−) as prevalence-independent measures of diagnostic test performance: LR+ = sensitivity / (1 − specificity); LR− = (1 − sensitivity) / specificity.

Abbreviations: AUC = area under the curve; CBD = corticobasal degeneration; CI = confidence interval; LR+ = positive likelihood ratio; LR− = negative likelihood ratio; MSA = multiple system atrophy; PD = Parkinson's disease; PPV = positive predictive value; PSP = progressive supranuclear palsy.

| **Table S7. Multiclass classification performance of the Random Forest model based on disease duration-adjusted [¹²³I]FP-CIT SPECT metrics (MSA-P and MSA-C combined as MSA)** | | | | |
| --- | --- | --- | --- | --- |
| **(A) Overall statistics** | | | | |
| Accuracy = 0.65 ± 0.05 Macro Balanced Accuracy = 0.68 Macro Sensitivity = 0.50 Macro Precision = 0.45 Macro F1 = 0.47 | | | | |
| **(B) Random Forest classification performance by diagnostic group** | | | | |
|  | **CBD** | **MSA** | **PD** | **PSP** |
| n | 43 | 49 | 487 | 127 |
| Balanced Accuracy | 0.638 | 0.657 | 0.738 | 0.683 |
| Sensitivity | 0.342 | 0.396 | 0.743 | 0.518 |
| Specificity | 0.933 | 0.919 | 0.733 | 0.847 |
| Precision (PPV) | 0.250 | 0.267 | 0.861 | 0.426 |
| F1 | 0.289 | 0.319 | 0.798 | 0.468 |
| Abbreviations: CBD = corticobasal degeneration; MSA = multiple system atrophy; PD = Parkinson’s disease; PSP = progressive supranuclear palsy. | | | | |

**Figure S2.** A) Distilled decision tree summarising the main classification pathways of the Random Forest model trained on disease duration-residualized [¹²³I]FP-CIT SPECT semiquantitative metrics. A decision tree summarising the classification rules of the Random Forest model trained on residualized [¹²³I]FP-CIT SPECT semiquantitative metrics. The tree was fitted to the out-of-fold Random Forest predicted labels using the one-standard-error rule for complexity parameter selection (cp = 0.015) and a maximum depth of 4 nodes. Each node shows the predicted diagnostic class and the proportion of out-of-fold observations at that node. Thresholds are expressed in original [¹²³I]FP-CIT SPECT metric units, back-transformed at the median disease duration of the cohort (2.6 years) for clinical interpretability. (B) Variable importance ranking of [¹²³I]FP-CIT SPECT semiquantitative metrics based on the mean decrease in Gini impurity, averaged across all 100 cross-validation folds. Higher values indicate greater contribution to the overall classification performance of the Random Forest model.Plot of variables importance. It illustrates the ranking of the [¹²³I]FP-CIT SPECT semiquantitative metrics based on the Mean Decrease Gini index. Higher values indicate a greater contribution of the metric to the overall diagnostic accuracy of the Random Forest model. Abbreviations: AI = asymmetry index; (ant.) = anterior; (C) = contralateral hemisphere relative to the most affected side; CBD = corticobasal degeneration; (I) = ipsilateral hemisphere relative to the most affected side; MSA = multiple system atrophy; (post.) = posterior; PSP = progressive supranuclear palsy; SBR = specific binding ratio.

| **Table S8. Sensitivity analysis of RF performance using an alternative definition of hemisphere (left and right hemispheres)** | | |
| --- | --- | --- |
|  | **Primary model (ipsilateral/contralateral to the most affected side)** | **Sensitivity analysis (Left/Right hemispheres)** |
| Overall Accuracy (mean ± SD) | 0.64 ± 0.004 | 0.657 ± 0.005 |
| Macro Balanced Accuracy | 0.68 | 0.665 |
| Macro Sensitivity | 0.475 | 0.447 |
| Macro Precision | 0.406 | 0.409 |
| Macro F1 | 0.43 | 0.425 |
| Kappa | 0.344 | 0.342 |
| In the primary model, the ipsilateral hemisphere is defined as the side with the lower posterior putamen SBR. For sensitivity analysis, the anatomical left and right hemispheres are considered independently of SBR values. Both models implement fold-wise disease-duration residualization. The Synthetic Minority Over-sampling Technique (SMOTE) is applied exclusively within each training fold. Model performance is evaluated using 10×10 repeated stratified cross-validation.  **Figure S3.** A) Distilled decision tree summarising the main classification pathways of the Random Forest model trained on disease duration-residualized [¹²³I]FP-CIT SPECT semiquantitative metrics. A decision tree summarising the classification rules of the Random Forest model trained on residualized [¹²³I]FP-CIT SPECT semiquantitative metrics. The tree was fitted to the out-of-fold Random Forest predicted labels (fidelity = 81%) using the one-standard-error rule for complexity parameter selection (cp = 0.015) and a maximum depth of 4 nodes. Each node shows the predicted diagnostic class and the proportion of out-of-fold observations at that node. Thresholds are expressed in original [¹²³I]FP-CIT SPECT metric units, back-transformed at the median disease duration of the cohort (2.6 years) for clinical interpretability. (B) Variable importance ranking of [¹²³I]FP-CIT SPECT semiquantitative metrics based on the mean decrease in Gini impurity, averaged across all 100 cross-validation folds. Higher values indicate greater contribution to the overall classification performance of the Random Forest model.Plot of variables importance. It illustrates the ranking of the [¹²³I]FP-CIT SPECT semiquantitative metrics based on the Mean Decrease Gini index. Higher values indicate a greater contribution of the metric to the overall diagnostic accuracy of the Random Forest model. Abbreviations: AI = asymmetry index; (ant.) = anterior; (C) = contralateral hemisphere relative to the most affected side; CBD = corticobasal degeneration; (I) = ipsilateral hemisphere relative to the most affected side; MSA = multiple system atrophy; (post.) = posterior; PSP = progressive supranuclear palsy; SBR = specific binding ratio. | | |

**[¹²³I]FP-CIT SPECT acquisition protocol**

SPECT images were acquired on a Siemens Symbia T6 (Siemens Healthineers; n = 557, 79%) and a AXIS SPECT/CT (Picker International; n = 149, 21%), both with a dual-head rotating gamma camera and Fan-Beam collimator, using the same protocol. Image acquisition was started between 3 and 6 h after injection of 185 MBq of [^123^I]FP-CIT. To block thyroid uptake of free radioactive iodide, patients received 120 mg of potassium iodide orally at least 30 minutes before intravenous radiotracer injection. Medication management followed routine clinical practice. Potentially DAT-interfering medications were withheld on the day of the scan according to the [Summary of Product Characteristics for ioflupane (¹²³I](https://www.ema.europa.eu/en/documents/product-information/datscan-epar-product-information_en.pdf)). A total of 120 projections of 30s each over a 360° circular orbit was acquired on a 128×128 matrix (zoom 1.23; rotation radius of 14 cm) to build the 3D images (50 frames; frame duration: 30 s). During acquisition, an adjustable head holder was used to minimise patient head motion. All studies underwent visual quality control of projection data and reconstructed images to identify motion artifacts and technical issues affecting registration. When substantial motion led to failed registrations, acquisition was repeated when feasible. Reconstruction [voxel size: 3.9 × 3.9 × 3.9 mm (Symbia) // 3.2 × 3.2 × 3.2 mm (AXIS)] was performed by applying a filtered back-projection (FBP) reconstruction algorithm using a Butterworth filter [order: 5; cutoff frequency: 0.45(Symbia) // 0.42 (AXIS)] without attenuation correction.

The acquisition protocol was kept strictly standardized throughout the long study period; a minority of patients were scanned on a different scanner unit with marginally different reconstruction parameters. The large majority of images were acquired on the Symbia T6. A minority of patients were scanned on an AXIS SPECT/CT (Picker International) using the same acquisition protocol. Reconstruction algorithm and matrix size are equivalent for both devices, differing only slightly in terms of slice thickness. Due to the low spatial resolution characteristic of SPECT, we consider that this should not have a significant impact on quantification.

The proportion of patients scanned on the AXIS SPECT/CT was low and comparable across atypical parkinsonism groups (CBD: 7%, MSA-P: 3%, MSA-C: 8%, PSP: 6%), with no diagnostic group predominantly scanned on this unit. The higher proportion observed in the PD group (28%) reflects its substantially larger sample size (n = 487) rather than a systematic allocation pattern.

Next figure displays the individual-level distribution of the three [¹²³I]FP-CIT SPECT metrics that showed a very relevant discriminative performance in our study: the ipsilateral SBR of the posterior putamen (panel A), the caudate-to-posterior putamen ratio (panel B), and the sensorimotor-to-limbic ratio (panel C). Across all three metrics and all diagnostic groups, AXIS SPECT/CT patients (triangles) are distributed throughout the full range of values within each group, without systematic concentration at the upper or lower extremes of the distribution. This pattern indicates that the AXIS SPECT/CT did not produce systematically higher or lower values for any of the key discriminative metrics, further supporting the absence of directional scanner-related bias in the study.

To formally evaluate a potential residual impact of scanner differences on our results, we performed a sensitivity analysis including scanner unit as a binary covariate (AXIS SPECT/CT vs. Symbia T Series SPECT/CT, reference category) in all linear regression models. Results are summarized in Figure 3 below, which plots the p-values from the original model (x-axis) against those from the scanner-adjusted model (y-axis) for all pairwise contrasts vs. PD. Points along the diagonal dashed line indicate perfect stability between models. The large majority of contrasts (green dots) cluster near the diagonal, confirming that scanner adjustment had no impact on most comparisons (mean absolute change in Cohen's d: Δd = 0.057). Only three contrasts (red) crossed the significance threshold towards non-significance after scanner adjustment: AI anterior putamen CBD vs. PD (p = 0.042 → 0.125), SBR contralateral posterior putamen PSP vs. PD (p = 0.046 → 0.118), and SBR ipsilateral sensorimotor PSP vs. PD (p = 0.017 → 0.063). All three were borderline significant in the original model, with p-values close to the 0.05 threshold, and none represents a primary outcome of the study. The loss of significance after scanner adjustment likely reflects a reduction in residual degrees of freedom rather than true confounding, as the effect sizes showed minimal change. Notably, no contrasts gained significance after scanner adjustment. Thus, this overall pattern is inconsistent with systematic scanner-related confounding. The results of this analysis support our initial modeling decision, showing that the main findings are robust to scanner adjustment and that including the scanner unit does not alter the conclusions. Given the limited sample sizes in certain subgroups (particularly MSA-C, n = 12), adding further covariates would reduce degrees of freedom and increase the risk of overfitting, potentially compromising model stability. For the same reasons, formal harmonization approaches (e.g., ComBat) were not applied, as the small number of scans acquired on the second system and the absence of systematic protocol differences did not justify additional model complexity.

**Residualization sensitivity analysis**

The potential confounding by demographic and clinical variables needs explicit clarification. Regarding age and sex, we deliberately excluded these from residualization. In a predictive framework, differences in age and sex across diagnostic groups may reflect genuine biological characteristics of the syndromes rather than nuisance variation. For example, the older age typical for PSP and CBD, or known sex-related differences in dopaminergic function, carry diagnostic information. Removing this variance a priori could attenuate diagnostically relevant signals. This is supported empirically by Schmitz-Steinkrüger et al. (2021), showing that age and sex together account for less than 10% of inter-individual variability in [¹²³I]FP-CIT SBR, versus 60–78% explained by diagnostic group. However, disease duration warrants a different treatment. Unlike age and sex, disease duration introduces within-group variability in striatal DAT binding that is directly related to disease progression and largely independent of the diagnostic category. Without correction, the classifier may capture severity-related differences rather than diagnosis-specific imaging patterns. We therefore residualized all SPECT metrics for disease duration prior to modelling.

Moreover, we confirmed empirically that residualizing for age and sex did not improve, and in fact modestly reduced, classification performance across all metrics. The Table S8 summarizes a comparison of Random Forest classification performance across three residualization strategies: metrics residualized for disease duration only, metrics residualized for age, sex, and disease duration, and non-residualized original values. All of them were evaluated under the same 10-fold repeated stratified cross-validation framework. The model only residualized for disease duration achieved the best overall performance (accuracy = 0.643 ± 0.004, macro balanced accuracy = 0.681, macro F1 = 0.431), compared to 0.615 ± 0.009, 0.643 and 0.391, respectively, when age and sex were additionally included in the residualization. The model trained on non-residualized values showed intermediate overall accuracy (0.656 ± 0.006) but lower macro balanced accuracy (0.666) and macro F1 (0.420), reflecting the confounding influence of disease duration on within-group variability.

| **Table S9. Comparison of Random Forest classification performance across residualization strategies (10-fold repeated stratified cross-validation)** | | | | | | |
| --- | --- | --- | --- | --- | --- | --- |
| *Residualization strategy* | **Accuracy (mean ± SD)** | **Macro Balanced Accuracy** | **Macro Sensitivity** | **Macro Precision** | **Macro F1** | **Kappa** |
| Residualized: disease duration only | 0.643 ± 0.004 | 0.681 | 0.476 | 0.406 | 0.431 | 0.347 |
| Residualized: age + sex + disease duration | 0.615 ± 0.009 | 0.643 | 0.420 | 0.374 | 0.391 | 0.277 |
| No residualization (original values) | 0.656 ± 0.006 | 0.666 | 0.450 | 0.401 | 0.420 | 0.343 |

Taken together, these results confirm that disease duration is the only variable warranting residualization in this context. We therefore analyzed and residualized all SPECT metrics for disease duration prior to Random Forest modelling, using each metric's residuals from a regression on disease duration as classifier inputs.

*Reference:*

Schmitz-Steinkrüger H, Lange C, Apostolova I, et al. Impact of age and sex correction on the diagnostic performance of dopamine transporter SPECT. *Eur J Nucl Med Mol Imaging*. 2021;48(5):1445-1459. doi:[10.1007/s00259-020-05085-2](https://link.springer.com/article/10.1007/s00259-020-05085-2)
